# Severity Ranking of Missense and Frameshift Genetic Variants in SCD1 by In Silico and In Vitro Functional Analysis

**DOI:** 10.3390/nu16193259

**Published:** 2024-09-26

**Authors:** Hanna K. Susán, Gabriella Orosz, Veronika Zámbó, Miklós Csala, Éva Kereszturi

**Affiliations:** Department of Molecular Biology, Semmelweis University, H-1085 Budapest, Hungary; hanna.krisztina@gmail.com (H.K.S.); orosz.gabriella@phd.semmelweis.hu (G.O.); zambo.veronika@semmelweis.hu (V.Z.); csala.miklos@semmelweis.hu (M.C.)

**Keywords:** lipotoxicity, lipid metabolism, genetic variation, desaturase, missense and frameshift mutations, structural prediction

## Abstract

Background: A considerable proportion of the symptoms associated with excessive dietary intake can be attributed to systemic imbalances in lipid metabolism. The prominent toxicity of saturated fatty acids has been repeatedly demonstrated and sheds light on the protective role of stearoyl-CoA desaturase-1 (SCD1), the key enzyme for fatty acid desaturation. SCD1 protein expression is regulated at the levels of transcription, translation, and degradation. However, the modulating effect of the variability of the human genome must also be taken into account. Therefore, we aimed to ascertain whether natural missense or frameshift mutations in SCD1 (p.H125P, p.M224L, p.A333T, p.R253AfsTer7) could influence the expression, degradation, or function of the enzyme. Methods: In silico and in vitro experiments were conducted to comprehensively evaluate the consequences associated with each genetic variation, with the objective of using the results to propose a risk or severity ranking of SCD1 variants. Results: As anticipated, the p.R253AfsTer7 variant was identified as the most deleterious in structural, functional, and quantitative terms. The p.H125P variant also reduced the desaturation capacity of the enzyme in accordance with the predicted structural alterations and augmented degradation resulting from folding complications. This was aggravated by increased mRNA instability and accompanied by mild endoplasmic reticulum stress induction. The p.A333T protein exhibited an intermediate phenotype, whereas p.M224L showed no deleterious effects and even increased the amount of SCD1. Conclusions: In conclusion, the large-scale identification of genetic variations needs to be supplemented with comprehensive functional characterization of these variations to facilitate adequate personalized prevention and treatment of lipid metabolism-related conditions.

## 1. Introduction

The balance of lipid metabolism within the human body is contingent upon an adequate supply of diverse saturated (SFAs), monounsaturated (MUFAs), and polyunsaturated (PUFAs) fatty acids (FAs) of varying carbon chain lengths, tailored to the dynamic fluctuations in demand. However, excessive FA overload results in the abnormal accumulation of lipids, impaired cellular function, and even cell death in both adipose and non-adipose tissues [1,2,3]. This phenomenon is known as lipotoxicity. A number of studies conducted in diverse experimental settings indicate that SFAs and unsaturated FAs (UFAs) exert differential effects with respect to lipotoxicity. The highly detrimental effects of palmitate (a major SFA), which can be mitigated by the presence of oleate (a major MUFA), have been extensively validated through empirical evidence [4,5,6,7].

Shifts in the ratio of different SFAs and UFAs from the optimal to an extreme and inadequate availability to cells can lead to an imbalance in lipid homeostasis, which in turn has significant effects on numerous human organs, including the central nervous system (CNS), the digestive system, and the cardiovascular system. A growing body of evidence indicates that imbalances in the metabolism and levels of FAs are associated with the onset and progression of CNS disorders, including multiple sclerosis, Alzheimer’s disease, and Parkinson’s disease [8]. Furthermore, the role of FAs in inflammation and related chronic diseases is beyond doubt [9]. It has also been demonstrated that in inflammatory bowel disease (IBD), which is characterized by a chronic, recurrent inflammatory disorder of the gastrointestinal tract, SFAs exhibit pro-inflammatory and thus aggravating effects, while UFAs may protect against the disease [10]. Non-alcoholic fatty liver disease (NAFLD) is a pathological condition hallmarked by hepatic steatosis. This is ultimately a consequence of an excessive uptake of lipids, especially free FAs, to a level that outpaces the capacity of lipid secretion in the form of lipoproteins, and the accumulation leads to cell damage and even lipoapoptosis [11]. A number of studies have demonstrated a robust correlation of elevated SFA intake with cardiovascular disease [12], type 2 diabetes [13], and metabolic syndrome [14]. Furthermore, excessive body fat has been linked to an increased risk of certain human cancers, which may be due to the ability of cancer cells to redirect metabolic pathways to meet energy demands through FA metabolism [15].

Stearoyl-CoA desaturase-1 (SCD1) is a key enzyme in lipid metabolism and a pivotal player in the defense against lipotoxicity, as it provides the only route for the desaturation of SFAs and catalyzes the rate-limiting step in the synthesis of UFAs from SFAs. It is an endoplasmic reticulum (ER) membrane-bound protein that introduces the first double bond of cis configuration at the Δ9 position into saturated fatty acyl-CoA molecules, primarily palmitoyl-CoA and stearoyl-CoA, to produce palmitoleyl-CoA and oleyl-CoA, respectively [16]. The desaturase activity currently available to the cell is dependent on the level of SCD1 enzyme [17], which, in turn, is determined by transcriptional and post-transcriptional regulation of gene expression, including modulation of protein degradation. The activating (insulin, growth factors, glucose, sucrose, and cholesterol) and inhibitory (including leptin, glucagon, docosahexaenoic acid, and arachidonic acid) agents act through a variety of transcription factors (TFs) such as LXR, SREBP, PPAR, C/EBP, and TR [18]. In accordance with its metabolic function, SCD1 expression is regulated by different FAs in a complex manner. A highly conserved PUFA-sensitive region (PUFARE) has been identified in the upstream regulatory sequence of the *SCD1* gene, which allows a significant downregulation of the intracellular *SCD1* mRNA pool in the presence of linoleate [19,20]. It is also well established that SFAs and UFAs exert transcriptional activation and repression, respectively [21]. Given that SCD1 has a relatively short half-life, its intracellular concentration can be further regulated through the modulation of protein degradation [22].

Although the protein and mRNA levels and activity of SCD1 are clearly influenced by its natural genetic variations, relatively few of these have been characterized functionally or even included in association studies. It provides an elegant example of gene–environment interactions that both the leucine-containing version of the single missense polymorphism in SCD1 (rs2234970) and the minor allele of a base change in its promoter (rs1054411) increase the desaturation index in a FA-dependent manner [23,24]. However, other genetic variants are also known to affect *SCD1* expression through altering consensus regulatory sequences in the transcribed region. One variant of the 3′ untranslated region (UTR) (rs41290540) affects miRNA binding [25], while a given haplotype of two intronic mutations modifies the TF binding site [26], ultimately leading to reduced *SCD1* mRNA levels. Furthermore, other variants of SCD1 have been linked to alterations in a number of parameters that are closely related to lipid metabolism, including body mass index, risk of obesity and cardiovascular diseases, insulin sensitivity, inflammation, and serum cholesterol levels [25,27,28,29].

The present study aimed to examine the effect of the p.H125P, p.M224L, p.A333T, and p.R253AfsTer7 natural human genetic variations on the structure and function of SCD1. In silico predictions were corroborated by in vitro experiments, which demonstrated that the p.H125P variant, in conjunction with the frameshift mutant, proved to be the most deleterious. In addition to a reduction in protein levels, decreased mRNA stability, and a lower desaturation index, the p.H125P variant induced a minor ER stress due to increased aggregation propensity and protease sensitivity of the protein. The p.A333T variant exhibited an intermediate phenotype, while the p.M224L variant did not show any adverse effects on the parameters tested. Based on our results, we have established a severity ranking of SCD1 missense variants. Further analysis of these variants and their potential role in pathomechanisms may lead to a better understanding of the underlying causes of various diseases related to lipid metabolism.

## 2. Materials and Methods

### 2.1. Chemicals and Materials

The culture medium and supplements were procured from Thermo Fisher Scientific (Waltham, MA, USA). Actinomycin D, MG132, HEK293T, and SK-N-FI cells were procured from Sigma-Aldrich (St. Louis, MO, USA). The solvents methanol, acetyl-chloride, and n-hexane were procured from Merck (Rahway, NJ, USA). All chemicals utilized in this study were of analytical grade. All experiments and measurements were conducted using Millipore ultrapure water.

### 2.2. Web-Based Tools for In Silico Analysis

The studied missense genetic variations were selected from the NCBI database. Nucleotide numbering reflects cDNA numbering, with +1 corresponding to the A of the ATG translation initiation codon in the reference sequence. The overall impact of the SCD1 mutants was estimated using ten different online prediction tools (SIFT, PolyPhen-2, CADD, REVEL, MetaLR, MutationAssessor, Provean, MutPred2, Mutation Taster, and M-CAP [30,31,32,33,34,35,36,37,38,39]). All programs were accessed on 12 February 2023. The original ranges and thresholds of these tools are available in Supplementary Table S1. The three-dimensional structure of the human SCD1 enzyme was downloaded from the Protein Data Bank (4ZYO, http://www.pdb.org/pdb/home/home.do; accessed on 9 March 2022) [40]. The three-dimensional structures of the missense and frameshift variants were constructed using the I-TASSER online prediction program (https://zhanggroup.org/I-TASSER/, accessed on 22 April 2023) [41]. All images were created with the DeepView/Swiss-Pdb Viewer software, version 4.0.2 (www.expasy.org/spdbv/, accessed on 13 April 2018).

### 2.3. Expression Plasmid Constructs and Mutagenesis

The coding region of *SCD1* was amplified from HEK293T cDNA using iProof™ High-Fidelity DNA Polymerase (Bio-Rad, Hercules, CA, USA) in accordance with the manufacturer’s instructions. The purified DNA fragment was inserted into the pcDNA3.1(-) expression plasmid between the *Xho*I and *Hin*dIII restriction endonuclease cleavage sites. The missense variants under investigation, along with the Glu-Glu-tagged versions, were prepared by overlap extension PCR mutagenesis. The primers used for cloning and mutagenesis are listed in Supplementary Table S2. All mutations were confirmed by Sanger sequencing.

### 2.4. Cell Culture and Transfection

HEK293T (human embryonic kidney) and SK-N-FI (human neuroblastoma) cells were maintained in 12-well plates (1 × 10^6^ cells per well) in Dulbecco’s modified Eagle medium (DMEM) complemented with 10% fetal bovine serum and 1% penicillin/streptomycin mixture at 37 °C in a humidified atmosphere containing 5% CO_2_. The samples were transfected with 1 μg of pcDNA3.1(-)-SCD1 expression vectors using 3 µL of Lipofectamine 3000, which was augmented with 2 µL of P3000 (Invitrogen, Carlsbad, CA, USA) in 1 mL of DMEM. After 24 to 30 h following the transfection, the cells were collected and subjected to analysis.

### 2.5. Cell Treatments

In conducting the mRNA stability assay, the transfection medium was replaced after overnight incubation at 37 °C with 1 mL DMEM containing the mRNA synthesis inhibitor actinomycin D (5 µg/mL). The cells were then incubated for 0, 2, 3, 6, 9, or 24 h in 12-well plates.

For the proteasomal degradation assay, after 5 h of incubation at 37 °C, the transfection medium was replaced with 1 mL of DMEM containing a MG132 proteasome inhibitor at a final concentration of 2 µM, and the cells were incubated for a further 24 h. Non-transfected and pcDNA3.1(-) vector transfected cells were used as the control in all experiments.

### 2.6. Preparation of Cell Lysates

For GC-MS, cells were prepared as described elsewhere [42]. Briefly, 10 µL of cell homogenate corresponding to 50 µg protein was methylated in PTFE screw-capped Pyrex tubes. As the internal standard, 1 µg each of C13:0 and of C21:0 *iso* (Larodan AB, Solna, Sweden) was added in 50 µL of methanol. Derivatization was performed with 2 mL of methanolic acetyl-chloride (10%) and 500 µL of n-hexane at 95 °C for 1 h with vigorous shaking. After cooling to room temperature, 5 mL 6% K_2_CO_3_ solution was added. A total of 100 µL of the upper layer of n-hexane was transferred to a 500 µL auto-sampler vial and crimped.

Cell lysates were prepared for immunoblot analysis by removing the medium and washing the cells twice with PBS. A total of 100 µL RIPA lysis buffer (0.1% SDS, 5 mM EDTA, 150 mM NaCl, 50 mM Tris, 1% Tween 20, 1 mM Na_3_VO_4_, 1 mM PMSF, 10 mM benzamidine, 20 mM NaF, 1 mM pNPP, and protease inhibitor cocktail) was added to each well and the cells were scraped and briefly vortexed. After 15 min incubation at room temperature, the lysates were centrifuged for 5 min at maximum speed in a benchtop centrifuge at 4 °C to remove cell debris. The protein concentration of the supernatant was measured with a Pierce^®^ BCA Protein Assay Kit (Thermo Fisher Scientific, Waltham, MA, USA) and the samples were stored at −20 °C until further analysis.

Prior to total RNA isolation, the cells were rinsed twice with PBS, after which they were harvested in 350 µL RLT buffer (Qiagen, Hilden, Germany) containing 1% *β*-mercaptoethanol, in accordance with the manufacturer’s instructions. Subsequently, the samples were stored at −80 °C until further use.

### 2.7. GC-MS Analysis of Fatty Acid Profiles

Fatty acid methyl esters (FAMEs) were separated by a highly polar BPX70 column (10 m × 0.10 mm, 0.20 µm film thickness, SGE) with an 70% cyanopropyl polysilphenyl-siloxane stationary phase using a GC-2010 coupled to a GCMS-QP2010 detector from Shimadzu (Kyoto, Japan). The injection volume was 1 µL and a programmed temperature vaporizer (PTV) was employed in spilt mode 1:20 for 3 s, then switched to splitless mode for 1.3 min with a split ratio of 1:100 until the end of the run. The injection temperature was set to 72 °C; and after a 3 s delay, it was increased to 250 °C at a rate of 240 °C/min and held for 15 min. The liner was packed with CarboFrit^TM^ (Restek, Centre County, PA, USA). The temperature program was as follows: the initial oven temperature of 50 °C was maintained for 0.75 min, then increased to 155 °C at a rate of 40 °C/min, to 210 °C at a rate of 6 °C/min, and finally to 250 °C at a rate of 15 °C/min and held for 2 min. Helium was used as a carrier gas at a constant linear velocity of 50 cm/s. The detector temperature was kept at 250 °C. Characterization and identification of FAMEs was performed in the scanning mode. The quantification was performed in the selected ion monitoring (SIM) mode for the most intense fragments (saturated: *m*/*z* 74; monounsaturated: *m*/*z* 55; diunsaturated: *m*/*z* 67; polyunsaturated: *m*/*z* 79). Data acquisition and processing were performed using GC-MS Solution Software 2.32 (Shimadzu). Quantification was based on an external calibration using C21:0 *iso* as internal standard. C13:0 added to C21:0 *iso* at a constant ratio was used as a quality control [43].

### 2.8. Immunoblot Analysis

Aliquots of cell lysates (comprising 10–15 µg protein per lane) were subjected to SDS-PAGE on 12% Tris-glycine minigels and subsequently transferred onto Immobilon-P membranes (Millipore, Billerica, MA, USA). The primary and secondary antibodies were incubated overnight at 4 °C and for 1 h at room temperature, respectively. SCD1, Glu-Glu tag and Actin were detected with rabbit polyclonal antibodies (Cell Signaling, Danvers, MA, USA; 2438S, 2448S and 4970L, respectively), used at a dilution of 1:2000, followed by HRP-conjugated goat polyclonal anti-rabbit IgG (Cell Signaling, Danvers, MA, USA, 7074S) at a dilution of 1:2000. BiP, CHOP, and PDI were detected with mouse monoclonal antibodies (Santa Cruz Biotechnology, Dallas, TX, USA; sc-166490, sc-7351, and sc-7351, respectively), used at a dilution of 1:1000, followed by HRP-conjugated horse anti-mouse IgG (Cell Signaling, Danvers, MA, USA, 7076S) at a dilution of 1:2000. HRP was detected by C-DiGit^®^ Blot Scanner (LI-COR, Lincoln, NE, USA) using the SuperSignal West Pico Chemiluminescent Substrate (Thermo Fisher Scientific, Waltham, MA, USA). The original, unmodified blot images from the parallel experiments are available for download in the Supplementary Materials file.

### 2.9. RNA Isolation, cDNA Synthesis

Total RNA was isolated from transiently transfected HEK293T cells by using the RNeasy Plus Mini Kit (Qiagen, Germantown, MD, USA) according to the manufacturer’s protocol. The concentrations were determined using a NanoDrop1000 spectrophotometer (Thermo Fisher Scientific, Waltham, MA, USA). To evaluate the mRNA quality, the ratios of absorbance at 260/280 and 260/220 nm were determined. Additionally, agarose gel electrophoresis was employed to visualize bands corresponding to 28S and 18S rRNAs. The potential for DNA contamination was eliminated through the use of DNase I treatment, employing the RNAqueous^®^-4PCR Kit (Invitrogen, Carlsbad, CA, USA). cDNA synthesis was conducted using the SensiFAST^TM^ cDNA Synthesis Kit (Meridian Bioscience, Memphis, TN, USA) with 0.5 µg of DNA-free RNA. To exclude plasmid contamination, RT– samples were also prepared as controls.

### 2.10. qPCR Analysis

Quantitative PCR assay was conducted in a 20 µL final volume containing 5 µL of 20-fold diluted cDNA, 1× PowerUp^TM^ SYBR^TM^ Green Master Mix, and 0.5 µM of sense and antisense primers using a QuantStudio^TM^ 3 Real-Time PCR System (Thermo Fisher Scientific, Waltham, MA, USA). The *SCD1* and *XBP1s* sequences were amplified using primer pairs 5′-CTG GCC TAT GAC CGG AAG AAA-3′/5′-GAC CCC AAA CTC ATT CCA TAG G-3′ and 5′-CTG AGT CCG AAT CAG GTG CAG-3′/5′-ATC CAT GGG GAG ATG TTC TGG-3′, respectively. Additionally, *GAPDH* cDNA was also measured as an endogenous control using the primer pair 5′-GTC CAC TGG CGT CTT CAC CA-3′/5′-GTG GCA GTG ATG GCA TGG AC-3′. The initial step of the thermocycle involved denaturation and enzyme activation at 95 °C for a period of 2 min. Subsequently, 40 cycles were conducted, comprising 95 °C for 15 s, 55 °C for 15 s, and 72 °C for 1 min. Fluorescent signal measurement was performed during annealing. The reactions were carried out in triplicate, and a reaction mixture containing RNase-free water in lieu of template cDNA was used as a non-template control. The relative expression levels were quantified using the 2^−ΔCT^ method, whereby the ΔC_T_ values represent the difference between the C_T_-values of the endogenous control and the target genes.

### 2.11. Aggregation Analysis

Glu-Glu-tagged forms of the wild-type SCD1 and its variants p.H125P and p.A333T were expressed in HEK293T cells. After 24 h following transfection, the cells were collected, and the lysates were processed in accordance with the aforementioned methodology. Cell lysates (10 µg) were subjected to centrifugation at 17,000× *g* for 1 h at 4 °C. Subsequently, the distribution of the desaturase between the supernatant and pellet was analyzed by immunoblotting, utilizing an antibody directed against the Glu-Glu tag.

### 2.12. Trypsin Sensitivity Assay

The cell lysates (10 µg total protein) were supplemented with 0.1 M Tris-HCl (pH = 8.0) and 1 mM CaCl_2_ (final concentrations) and incubated with 100 nM human cationic trypsin (final concentration) in 20 mL volumes for 0, 15, 30, and 60 min. Desaturase degradation was followed by immunoblot analysis using an anti-Glu-Glu tag antibody.

### 2.13. Statistical Analysis

Immunoblots were quantified using the Image Studio^®^ 5.2 software (LI-COR Biotechnology, Lincoln, NE, USA), with results expressed as relative band densities normalized to Actin as a reference. The mean values ± S.D. for relative band densities and mRNA levels are presented in the diagrams and were compared by ANOVA with Tukey’s multiple comparison post hoc test using the GraphPad Prism 6.0 software (GraphPad Software, Boston, MA, USA). A *p*-value of less than 0.05 was considered to indicate a statistically significant difference.

## 3. Results

### 3.1. Predicted Impact of Natural Genetic Variants on SCD1

**Figure 1:**
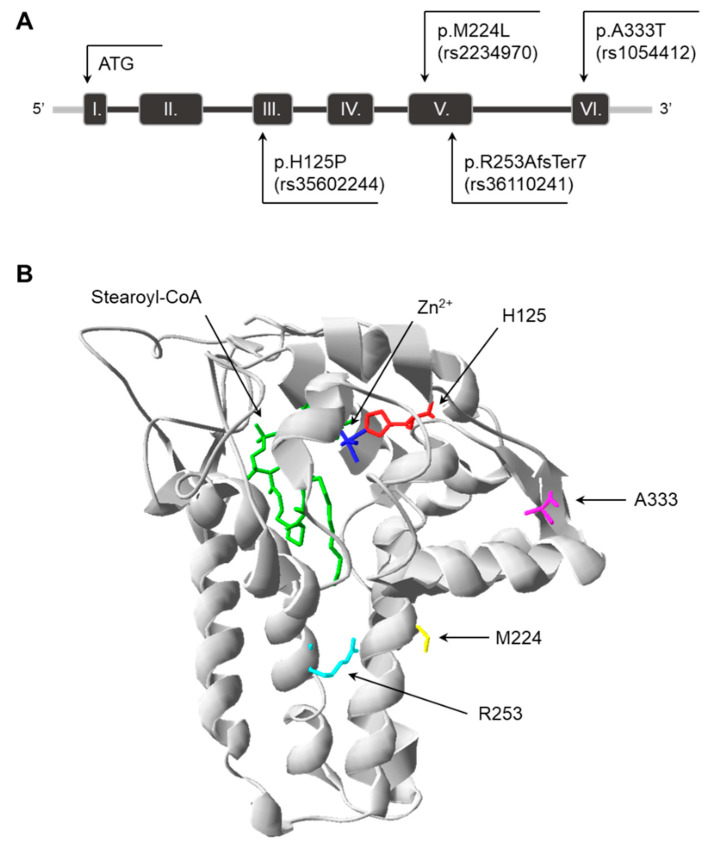
Localization of genetic variations in the SCD1 gene and protein. (**A**): Exons are numbered with Arabic numerals; the rs ID of each variant is also indicated. (**B**): The crystal structure of the wild-type human SCD1 was obtained from the Protein Data Bank (file 4ZYO). The image was rendered with DeepView/Swiss-Pdb Viewer version 4.0.2. The positions of the amino acids affected by genetic variations were indicated by different colors (red, yellow, light blue, and pink), and one of the catalytic Zn^2+^ ions was highlighted in dark blue. The substrate stearoyl-CoA is also shown in the active site in green.

At the start of the study, four natural genetic variants affecting the coding region of *SCD1* were selected. The p.H125P (c.374A>C, rs35602244) and p.A333T (c.997G>A, rs1054412) variants are located at the beginning of the third and sixth exons of the gene, respectively (Figure 1A), and result in amino acid substitutions on the cytosolic globular surface of the enzyme (Figure 1B). The p.M224L variant in the fifth exon (Figure 1A) is the only known missense polymorphism of SCD1 (c.670 A>C, rs2234970), which changes the 224th methionine of the third transmembrane domain (TM) to leucine (Figure 1B). The fifth exon frameshift variant, p.R253AfsTer7 (c.752_753insT, rs36110241), affects the fourth TM of the SCD1 protein (Figure 1).

**Figure 2:**
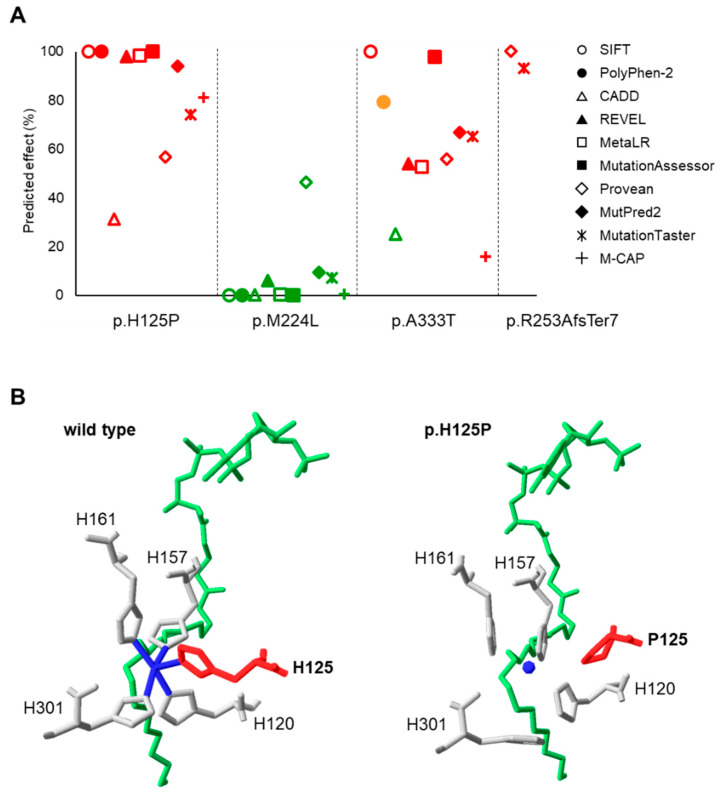
Prediction of the effect of SCD1 genetic variants. (**A**): Diagrammatic representation of the overall effect of SCD1 variations predicted by ten different prediction tools. The scale of impact ranges from 0 to 100%, with higher values indicating more adverse effects. Red, orange, or green markers represent a damaging, intermediate, or benign predicted effect, respectively, according to the classification of the corresponding program. (**B**): Predicted structural effect of p.H125P variant. The crystal structure of the wild-type human SCD1 was downloaded from the Protein Data Bank (file 4ZYO). The 3D structure of p.H125P SCD1 variant was predicted by I-TASSER. Images were generated with DeepView/Swiss-Pdb Viewer version 4.0.2. Amino acid 125 is indicated in red, Zn^2+^ ion in dark blue, and stearoyl-CoA in green.

The overall effect of the four SCD1 variants on the protein was assessed using ten different online prediction programs (SIFT, PolyPhen-2, CADD, REVEL, MetaLR, MutationAssessor, Provean, MutPred2, Mutation Taster, and M-CAP), of which only two (Provean and MutationTaster) were suitable for frameshift mutants. As some of the programs apply different scaling or set different thresholds for the harmfulness of a mutation, the scores generated by each tool were converted to a common scale for easier comparison (Figure 2A). The original and converted data are presented in Supplementary Table S1. The p.H125P mutation was predicted to be deleterious by all programs, as was the frameshift variant. In contrast, the overall predictions of p.M224L unanimously found this polymorphism to be benign. The p.A333T variant exhibits a more diverse pattern, with eight tools predicting deleterious effects, CADD indicating a benignity and PolyPhen-2 suggesting an intermediate phenotype (Figure 2A).

The 3D prediction by the I-TASSER program indicated that none of the SCD1 mutants tested lead to a loss of overall protein structure. However, different degrees of structural changes were observed in all cases (Supplementary Figure S1). As anticipated, the p.R253AfsTer7 variant results in the creation of a STOP codon in the post-insertion region, leading to truncation of the C-terminal portion of the protein from the middle of the fourth TM domain (Supplementary Figure S1D). Since this missing peptide sequence is an essential component of the substrate-binding region, it seems plausible that its absence results in the insufficient substrate-binding capacity of the enzyme. The p.M224L and p.A333T variants are located at a distance from the substrate binding site and the metal ions required for catalysis. Apart from minor structural rearrangements, these variants do not significantly affect the protein structure (Supplementary Figure S1B,C). Although the overall spatial structure of the p.H125P mutant is very similar to the wild type (Supplementary Figure S1A), the histidine → proline substitution at position 125 may significantly reduce the ability of this variant to retain catalytically crucial Zn^2+^ in coordination binding (Figure 2B).

### 3.2. Altered Cellular Fatty Acid Profile of SCD1 Missense and Frameshift Variants

**Figure 3:**
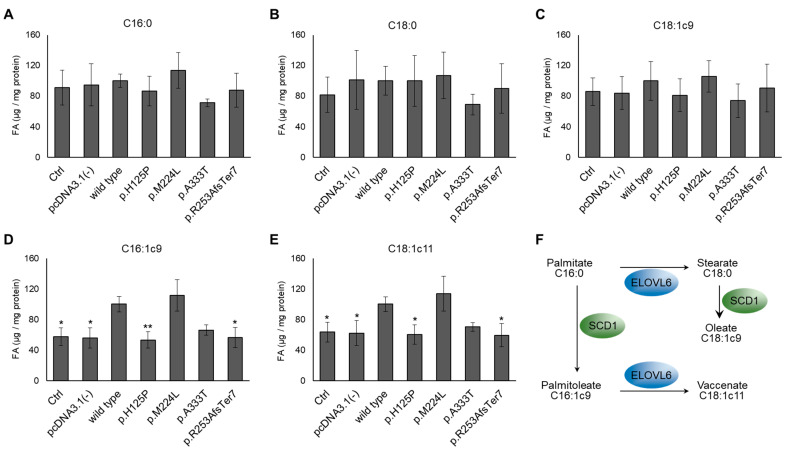
Desaturated profile upon overexpression of p.H125P, p.M224L, p.A333T, and p.R253AfsTer7 SCD1 variants. Transiently transfected HEK293T cells were collected, and the FA profile was measured by GC-MS after sample preparation, as described in the *Materials and Methods* section. The quantities of (**A**) palmitate, (**B**) stearate, (**C**) oleate, (**D**) palmitoleate, and (**E**) vaccenate were normalized to the total protein content of the samples and are presented as mean values ± S.D.; n = 3. The given FA content of the wild-type SCD1 was considered to be 100%. Statistical analysis was performed using the Tukey–Kramer multiple comparisons test. * *p* < 0.05; ** *p* < 0.01. (**F**): The enzymes interconverting the highlighted FAs. ELOVL6: fatty acid elongase 6; Ctrl: control.

The composition of the FA content of HEK293T cells transiently transfected with SCD1 mutants was analyzed by measuring a wide panel of saturated, monounsaturated, and polyunsaturated FAs in the range of 10 to 24 C atoms using GC-MS. The raw data can be found in Supplementary Table S3 for all detected FAMEs. Transfection with an empty vector did not alter the intracellular FA profile (Figure 3, Supplementary Figure S2, Supplementary Table S3). The overall desaturation intensity, reflecting SCD1 activity, was characterized by the calculated ratio of the sum of two major monounsaturated FAs and the sum of two major saturated FAs, i.e., (palmitoleate (C16:1c9) + oleate (C18:1c9))/(palmitate (C16:0) + stearate (C18:0)). The overall SCD1-dependent desaturation rate did not differ significantly between cells expressing each mutant version of the enzyme and the wild type; however, it is worth noting that there was a tendency towards a decrease in the p.H125P mutant (Supplementary Figure S2).

Palmitate (C16:0), the primary product of de novo FA synthesis, is intensely elongated to stearate (C18:0) by ELOLV6 (Figure 3F). They are the two main substrates of SCD1, which converts them to palmitoleate (C16:1c9) and oleate (C18:1c9), respectively. The amounts of these FAs were analyzed separately in the cells expressing different mutants or the wild-type enzyme. No reduction in the amount of the two substrates or elevation in the amount of oleate was detected when comparing the cells expressing wild-type SCD1 with the control (untransfected) or empty vector transfected samples (Figure 3A–C).

In contrast, the amount of palmitoleate, the other major product of SCD1, shows significant and mutation-dependent alterations (Figure 3D). The expression of wild-type SCD1 alone resulted in a nearly twofold increase in palmitoleate levels compared to both the untreated control and the empty vector transfected samples. The amount of palmitoleate detected in the presence of p.M224L was slightly elevated above that found in the wild type, in accordance with previously published results [23], and p.A333T showed a 33% lower level, although these differences were not significant. The palmitoleate content of cells expressing p.H125P or p.R253AfsTer7 was significantly reduced to the level of the controls, i.e., approximately half of the wild type, which is not unexpected given the structural predictions (Figure 2, Supplementary Figure S1). This pattern was also reflected in the absolute amount of vaccenate derived from the ELOVL6-mediated elongation of palmitoleate (Figure 3E,F). The p.M224L and p.A333T mutations exhibited a tendency to increase and decrease the amount of vaccenate, respectively, although these changes were not statistically significant. The p.H125P variant and the frameshift mutant, however, again showed a statistically significant reduction in the amount of this UFA (Figure 3E).

The observed alterations in FA composition resulting from SCD1 missense and frameshift mutations may be attributed to changes in the amount of the intracellularly available desaturase enzyme. To test this assumption, we studied the protein and mRNA levels, as well as the intracellular fate and degradation of mutant forms.

### 3.3. Modulating Effect of SCD1 Mutations on Intracellular Protein Levels

**Figure 4:**
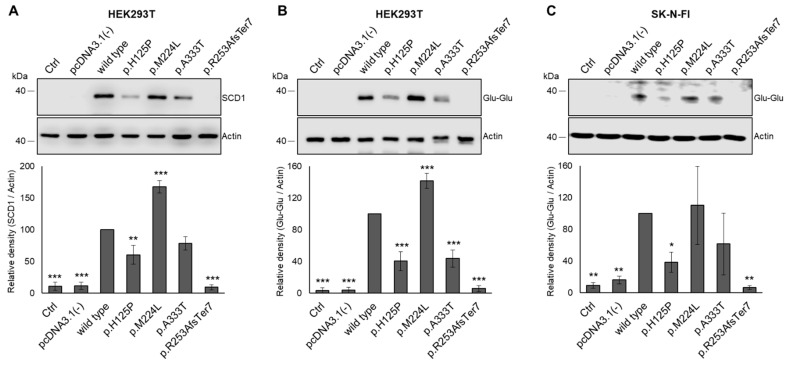
Expression of SCD1 variants in transiently transfected HEK293T and SK-N-FI cells. The HEK293T and SK-N-FI cells were collected and subjected to the requisite processing 24 h following the transfection. Aliquots of cell lysates (10 µg protein) were loaded onto a 12% SDS–polyacrylamide gel and, after electrophoresis, transferred to an Immobilon-P membrane. SCD1 was detected using either anti-SCD1 (**A**) or anti-Glu-Glu tag (**B**) antibody in HEK293T cells. The amount of the Glu-Glu-tagged SCD1 mutant proteins was also assessed in SK-N-FI cells (**C**). Actin was used as a loading control. Representative immunoblots of three independent experiments are shown. Band intensities were quantified by densitometry, and the SCD1/Actin ratios are presented as bar graphs. The quantity of the wild-type SCD1 protein was considered to be 100%. Data are shown as mean values ± S.D. Statistical analysis was performed with the Tukey–Kramer multiple comparisons test. * *p* < 0.05; ** *p* < 0.01; *** *p* < 0.001. Ctrl: control.

SCD1 protein content was analyzed in HEK293T cells transiently transfected to express the wild-type and p.H125P, p.M224L, p.A333T, and p.R253AfsTer7 human SCD1 variants. Under given experimental conditions, no endogenous SCD1 protein was detected in the non-transfected and empty vector transfected control cells, and a massive overexpression could be observed in the wild-type SCD1 transfected cells by immunoblot (Figure 4). To rule out the possibility that altered band densities are due to altered affinity of the antibody, SCD1 variants were also expressed with a C-terminal Glu-Glu epitope tag (Figure 4B). The amount of the tagged proteins exhibited a similar pattern to that of the corresponding untagged versions (Figure 4A,B).

Consistent with our earlier studies, significantly higher intracellular protein levels were observed for the p.M224L polymorphic variant compared to the wild-type protein, which served as a positive control [23]. In accordance with the lower desaturase capacity (Figure 3) and the predicted structural anomalies (Figure 2), transfection with p.H125P led to a significant decrease in the cellular SCD1 levels, whether untagged (60%, Figure 4A) or tagged (40%, Figure 4B). The p.A333T protein is also characterized by lower protein levels, although this was only significant for the tagged version (Figure 4B). As might be expected, the frameshift genetic variant was not detectable at all.

To test the potential cell specificity of the above phenomena, the experiments were repeated using another cell line and the tagged constructs. The expression pattern observed in transfected SK-N-FI cells matched well with that seen in HEK293T, i.e., the levels of p.H125P and p.R253AfsTer7 proteins were also notably reduced compared to the wild type (Figure 4C). Although the levels of p.M224L were increased and p.A333T decreased, these changes were not statistically significant due to the large standard deviation in the neuroblastoma cell samples.

### 3.4. Altered mRNA Levels and Stability of p.H125P and p.A333T SCD1 Variants

**Figure 5:**
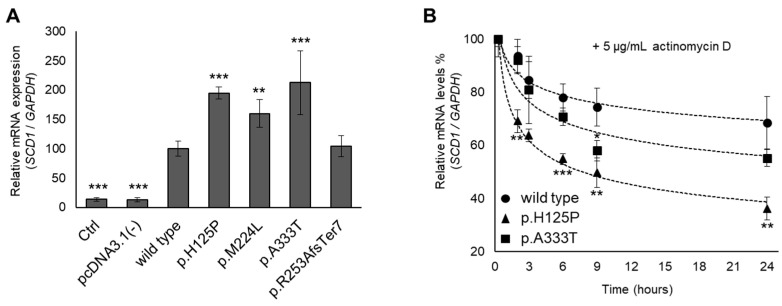
mRNA level and stability of *SCD1* variants. (**A**): mRNA levels were measured in transiently transfected HEK293T cells. (**B**): After 24 h since transfection, HEK293T cells were treated with actinomycin D (5 µg/mL) for 0, 2, 3, 6, 9, or 24 h. The samples were harvested and prepared as described in the Materials and Methods. qPCR was carried out using *GAPDH* and *SCD1* sequence-specific primers, as indicated in the Materials and Methods and Supplementary Table S2. The diagram depicts the outcome of at least three independent assessments, wherein the quantity of the wild-type *SCD1* mRNA was considered to be 100%. Statistical analysis was performed with the Tukey–Kramer multiple comparisons test. Data are shown as mean values ± S.D. ** *p* < 0.01; *** *p* < 0.001. Ctrl: control.

*SCD1* mRNA levels were compared by qPCR in the samples from the transfected HEK293T cells. Amplification in the RT– controls reached the threshold 7–10 cycles later than in the transfected samples. Endogenous mRNA expression can therefore be considered negligible, i.e., 3–4 orders of magnitude lower than overexpression. All three missense mutations yielded a significant increase in mRNA levels, 1.95-fold, 1.60-fold, and 2.13-fold for p.H125P, p.M224L, and p.A333T, respectively (Figure 5A). The mRNA with a frameshift mutation was detectable at levels comparable to the wild type.

The increased mRNA stability of p.M224L has been described previously [23]. We also assessed the intracellular degradation of p.H125P and p.A333T mRNA variants in transfected HEK293T cells using actinomycin D as a transcription inhibitor and qPCR analysis. A significant difference between p.H125P and wild-type *SCD1* was evident as soon as 2 h after transcription (Figure 5B). The wild-type *SCD1* transcript was reduced by only 6% from the initial amount, whereas the p.H125P variant already dropped by 31%. The difference remained statistically significant throughout the experiment. Two-thirds of the wild-type *SCD1* mRNA was still present after 24 h, when the p.H125P variant was already reduced to one-third. The p.A333T mutation showed an intermediate degradation rate, representing lower mRNA levels than the wild-type allele, yet higher mRNA stability than the p.H125P variant. This was found to be significant for the 9 h samples (Figure 5B).

Our results show that the accelerated mRNA degradation in the case of the p.H125P and p.A333T variants is compensated by more intense synthesis, and mRNA expression is still higher overall. Therefore, no explanation for the lower protein levels observed in the two variants was found at the mRNA level, so we looked further at protein stability.

### 3.5. Proteasome Inhibitor Prevents Intracellular Degradation of p.H125P and p.A333T SCD1 Variants

**Figure 6:**
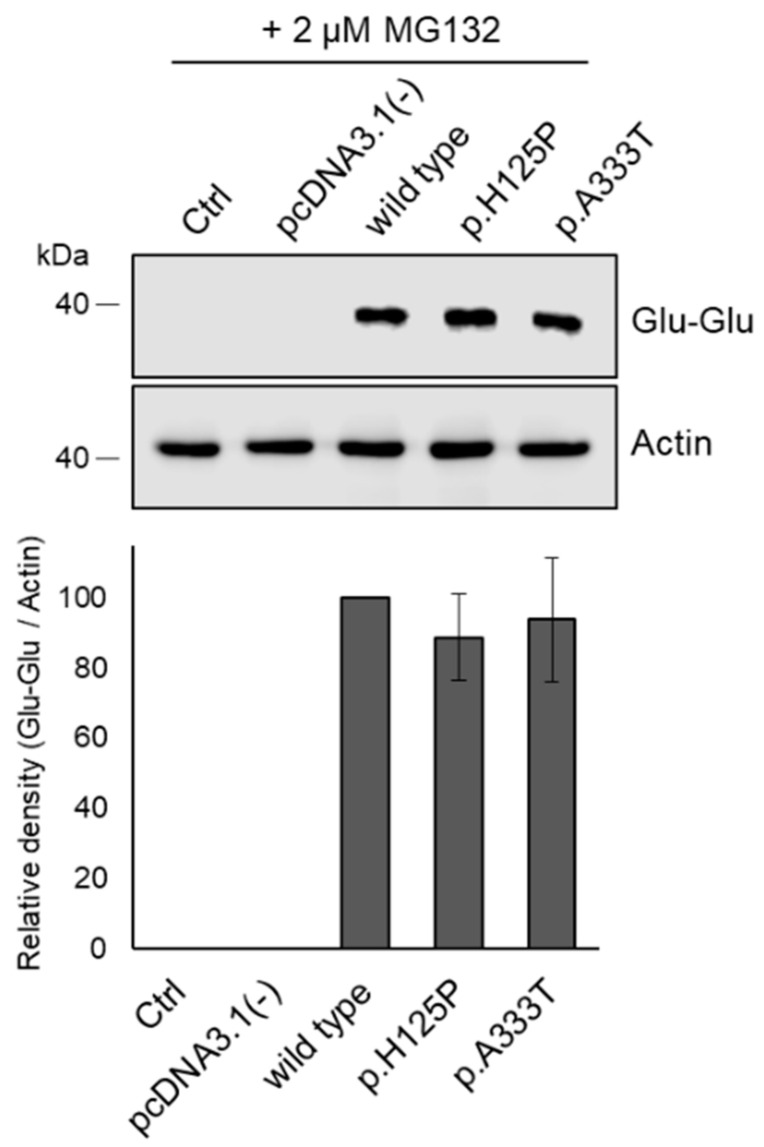
Effect of proteasome inhibitor on the levels of SCD1 variants. HEK293T cells were transiently transfected with wild-type, p.H125P, and p.A333T SCD1 constructs and treated with 2 µM MG132 for 24 h. Aliquots of cell lysates (10 µg) were loaded onto a 12% SDS–polyacrylamide gel and, after electrophoresis, transferred to an Immobilon-P membrane. SCD1 was detected using an anti-Glu-Glu tag antibody. Actin was used as a loading control. Representative immunoblots of three independent experiments are shown. Band intensities were quantified by densitometry, and the SCD1/Actin ratios are presented as bar graphs. The quantity of the wild-type SCD1 protein was considered to be 100%. Data are shown as mean values ±S.D. Statistical analysis was performed with the Tukey–Kramer multiple comparisons test. Ctrl: control.

The degradation of the p.H125P and p.A333T SCD1 proteins was investigated using a cell-permeable proteasome inhibitor, MG132, applied at the lowest effective concentration (2 µM) previously reported [44]. The treatment of transfected HEK293T cells with MG132 resulted in the complete abolition of the observed difference in protein levels, with wild-type and mutant SCD1 variants being expressed to a similar extent (Figure 6).

### 3.6. Increased Intracellular Aggregation and Protease Sensitivity of p.H125P and p.A333T SCD1 Variants

**Figure 7:**
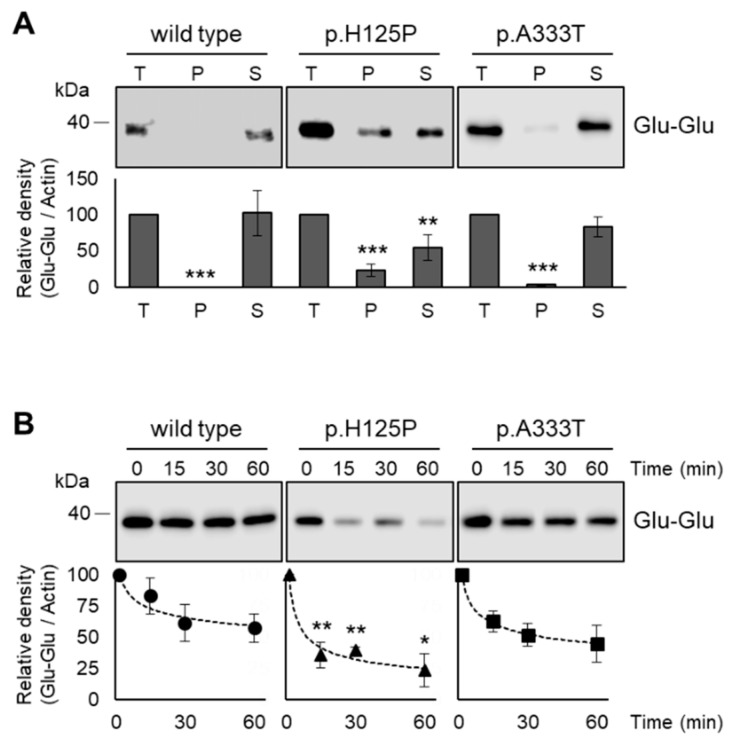
Intracellular aggregation (**A**) and protease sensitivity (**B**) of wild-type, p.H125P, and p.A333T SCD1. Glu-Glu-tagged forms of wild-type and mutant SCD1 were expressed in HEK293T cells. At 24 h after transfection, cells were harvested, and lysates were prepared, as described in the Materials and Methods. Aliquots of samples (10 µg) were loaded onto a 12% SDS–polyacrylamide gel and, after electrophoresis, transferred to an Immobilon-P membrane. SCD1 was detected using an anti-Glu-Glu tag antibody. Representative immunoblots of three independent experiments are shown. Band intensities were quantified by densitometry and are presented as bar graphs. The quantity of wild-type SCD1 protein was considered to be 100%. Data are shown as mean values ± S.D. Statistical analysis was performed with the Tukey–Kramer multiple comparisons test. * *p* < 0.05; ** *p* < 0.01; *** *p* < 0.001; Ctrl: control.

Protein degradation and hence overall protein expression level can be largely affected by the aggregation of incorrectly folded proteins. To test whether this factor plays a determining role in the intracellular fate of p.H125P and p.A333T SCD1 proteins, cell lysates of transfected HEK293T cells expressing Glu-Glu-tagged wild-type, pH125P, or p.A333T SCD1 were subjected to high-speed and long-time centrifugation, and the SCD1 contents of the pellet and the supernatant were compared by immunoblot (Figure 7A). As expected, the wild-type protein did not sediment and the total amount remained in the supernatant, indicating a lack of detectable aggregation. The sedimentation of p.A333T SCD1 was minimal, with most of the protein retained in the supernatant. However, a significant portion (23%) of the p.H125P protein was recovered from the pellet, indicating a remarkable susceptibility of this variant to aggregation in the cell.

To provide further evidence for the partially misfolded state of the p.H125P mutant, cell lysates were subjected to trypsin digestion for 0, 15, 30, and 60 min, and the protease sensitivity of the wild-type and p.H125P desaturases was compared (Figure 7B). Following a 15 min incubation period, a substantial proportion (64%) of the p.H125P variant was digested by trypsin, whereas wild-type SCD1 exhibited a much greater resistance to protease cleavage, with as little as 17% degradation. This statistically significant difference in the rate of fragmentation persisted until the completion of the one-hour experiment, leaving 57% and 23% of the wild type and p.H125P, respectively. As expected, the p.A333T variant showed an intermediate phenotype, displaying a slightly reduced trypsin resistance at all time points compared to wild type, but not reaching the sensitivity levels observed in the p.H125P protein.

### 3.7. ER Stress in HEK293T Cells Expressing Genetic Variants of SCD1

**Figure 8:**
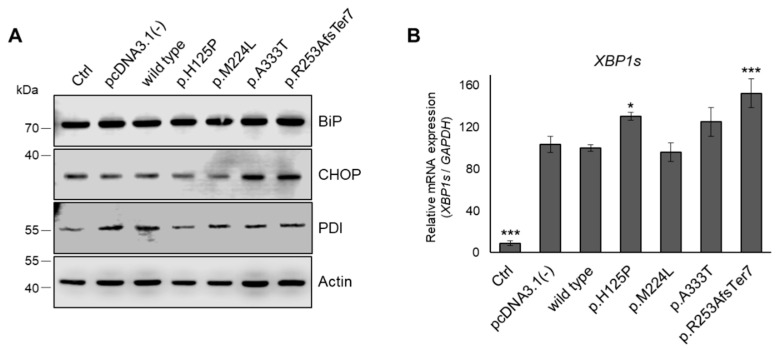
ER stress markers in HEK293T cells expressing wild-type or mutant SCD1 proteins. (**A**): BiP, CHOP, and PDI protein levels in cell lysates (10 µg total protein) were analyzed by immunoblotting, as described in the Materials and Methods. Actin was used as loading control. Representative immunoblots of three independent experiments are shown. (**B**): The extent of *XBP1* splicing was determined by qPCR. Expression of *GAPDH* was measured as a reference control, as described in the Materials and Methods. This Figure shows the results of three independent measurements, where the amount of *sXBP1* mRNA in wild-type SCD1 samples was considered to be 100%. Statistical analysis was performed with the Tukey–Kramer multiple comparisons test. * *p* < 0.05; *** *p* < 0.001; Ctrl: control.

The retention and accumulation of misfolded proteins in the ER can lead to ER stress, which in turn triggers the unfolded protein response [45]. To investigate whether the SCD1 variants induce ER stress, we assessed the levels of BiP, CHOP, and PDI. As illustrated in Figure 8A, no elevation in these ER stress markers was observed for any of the SCD1 variants. The spliced form of *XBP1* mRNA (*sXBP1*) was also monitored by qPCR (Figure 8B). Transfection alone resulted in a tenfold increase in *sXBP1* levels, and the elevation was significantly greater (by approximately 30%) in the case of p.H125P, and even more so (by 53%) for p.R253AfsTer7.

## 4. Discussion

Although human SCD1 has been demonstrated to be a key enzyme in lipid metabolism and is likely to be involved in the pathogenesis of numerous common diseases, its natural genetic variants have been poorly studied. However, according to the NCBI SNP database, the gene itself hosts more than 7000 single nucleotide polymorphisms (SNPs), nearly two-thirds of which are intronic variants. The number of SNPs in the regulatory region is over 1000, while missense variants account for 3% of the total SNPs. Only one of the latter has been analyzed thus far (p.M224L, rs2234970), and it has been reported that the leucine-containing variant is distinguished by its slower protein degradation rates and enhanced mRNA stability. The consequently elevated expression levels of the 224L SCD1 enzyme also lead to a marked increase in the FA desaturation index. Although the association of this missense genetic variation with type 2 diabetes has not been conclusively proven, it has been demonstrated to enhance cardiometabolic risk [46].

The 5′ and 3′UTRs of *SCD1* are also polymorphic, although these genetic variations have not been extensively studied to date. Four SNPs (rs1054411, rs670213, rs2275657, and rs2275656) have been identified in the 5′ regulatory region of *SCD1* and demonstrated not to affect promoter activity per se. Moreover, it has been evidenced that rs1054411 enhances transcription in a FA-dependent manner, while the same polymorphism has been shown to reduce gene expression through ETS1 TF-binding [24]. In contrast, several genetic variations in the 3′UTR have been associated with specific conditions affecting lipid metabolism. The minor allele of rs7849 polymorphism has been demonstrated to result in diminished SCD1 enzyme activity, consequently leading to a reduced desaturation index [28]. Moreover, this allele has been found to be positively associated with a lower body mass index and increased insulin sensitivity [27], as well as a reduced risk of developing obesity [28]. Another SNP in the 3′UTR of *SCD1*, rs41290540, has been shown to modify the miR-498 miRNA binding site. This binding site is abolished in the minor allele, thus preventing miR-498 from regulating SCD1 levels. Additionally, this polymorphism has been associated with coronary artery disease and lower serum cholesterol levels [25].

The intronic rs10883463 SNP, which also negatively affects the desaturation activity [28], is associated with a predisposition to obesity and with the degree of insulin resistance [27], while rs1502593 genetic variation has been associated with metabolic syndrome [47]. Despite the fact that the functional role of intronic variants is often not, or only with difficulty, investigated due to their location, Pan and colleagues have presented an elegant study demonstrating the role of two *SCD1* intronic SNPs in transcriptional regulation. Two polymorphisms (rs55710213 and rs56334587) in intron 5 of *SCD1* affect the TF binding site for HNF4A, a key regulator of lipid and carbohydrate metabolism. The haplotype of the minor alleles of these two SNPs has been shown to significantly reduce the probability of HNF4A-binding to DNA, and therefore reduce *SCD1* gene expression [26].

The exact role of genetic variants in non-coding regions is often challenging to determine by functional molecular biology, and thus their impact is mainly addressed by association studies. The minor alleles of rs508384 and rs2167444 *SCD1* polymorphisms have been demonstrated to be associated with reduced desaturase activity and increased risk of obesity and insulin resistance [27,28]. Furthermore, the minor alleles of both variants have been linked to elevated ApoB-48 levels and increased risk for other cardiometabolic conditions, including an unfavorable lipid profile, elevated systolic blood pressure, and oxidative stress [48]. The rs1393492 SNP is prevalent in individuals with metabolic syndrome [49], and the rs2060792 variant may contribute to the development of inflammatory conditions [29].

**Figure 9:**
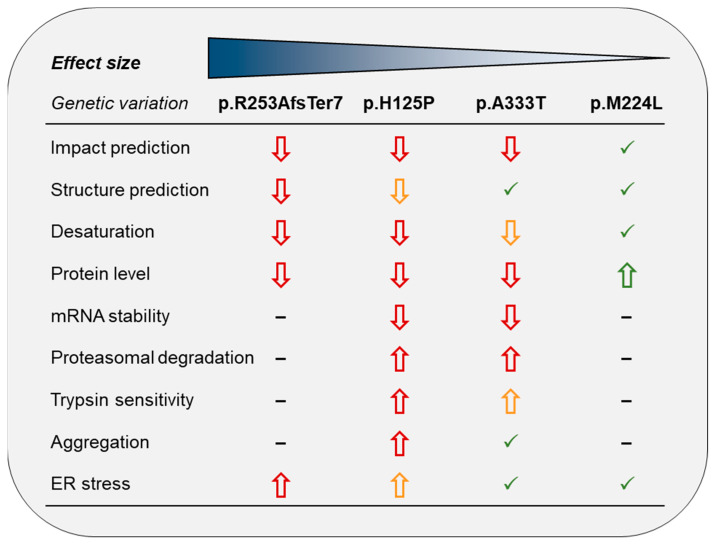
Summary of the scalable functional impact of SCD1 genetic variants. The colors red, orange, and green indicate harmful, moderate, and benign effects, respectively. The direction of the arrow represents the direction of change. Checkmarks indicate no difference from the wild-type SCD1, while dashes indicate no data.

The meticulous analysis of the vast amount of genetic variation present in whole-genome sequencing data and the assessment of their relevance remain a significant scientific challenge. The prediction of functional and/or regulatory impacts of various mutations using in silico approaches represents an important step toward the identification of variants that are functionally significant and/or clinically relevant [50]. Nevertheless, it should be noted that none of these prediction methods are infallible. For instance, the reliability of effect prediction is extremely low for missense variants involving poorly conserved regions of proteins [51]. It is imperative to emphasize the necessity of caution when relying on algorithms that predict pathogenicity to make a genetic diagnosis of patients. Furthermore, there is clearly a need for the development of alternative in vitro or in vivo models for the functional validation of natural genetic variants in a more accurate and efficient manner, regardless of whether they are classified as pathogenic or benign [52]. Along these lines, the present study commenced with an in silico effect analysis of three missense and one frameshift variants of SCD1, the results of which were either refuted or confirmed by subsequent in vitro experiments. In accordance with the principles of designing scalable functional assays [53], a number of parameters were tested, the cumulative results of which are presented in Figure 9.

The frameshift variant had the greatest in silico predicted overall effect and a major impact on the 3D protein structure. However, the histidine → proline substitution at position 125 was also found to be significantly deleterious, probably due to the loss of a histidine that is critical for enzyme function (Figure 1 and Figure 2, Supplementary Figure S1). Consistently, all the parameters examined (Figure 9) were negatively affected by these variants. It is noteworthy that the reduction in desaturase activity was only discernible for one of the metabolic products (palmitoleate) and its derivative (vaccenate), and no difference was observed in the amount of oleate or in the overall desaturation index (Figure 3, Supplementary Figure S2). The proportions of palmitoleate and vaccenate were relatively low in the cells (Supplemental Table S3) in comparison to many other components, which may be the reason for a more detectable change in their amounts. It should be noted that the cells also had their own wild-type SCD1 enzyme, and the assay attempted to detect differences against endogenous SCD1 expression. On the other hand, it is possible that the mutation-specific differences in the amount of oleate could not be observed because the palmitate affinity of ELOVL6 exceeded that of SCD1, masking the changes.

The reduced desaturation by p.H125P and, to a lesser extent, by p.A333T enzymes may be a consequence of lower intracellular protein expressions with these genetic variations. The mutations resulted in a partial impairment of protein folding, increased exposure of the enzyme to protease cleavage, and enhanced aggregation, which could target the protein towards proteasomal degradation. Furthermore, the misfolding and enhanced aggregation of the protein may disturb the ER function, thereby increasing the likelihood of ER stress (Figure 9). Such effects of missense mutations are considered to be quite general, and similar impacts have been described across a broad spectrum of protein functions [44,54,55,56]. The two most damaging variants of SCD1 did not result in detectable increase in levels of the three long-term ER stress markers; the ER chaperones and foldases, BiP and PDI; and the pro-apoptotic TF, CHOP, under our experimental conditions, including relatively short exposures. Nevertheless, both increased the levels of *sXBP1* mRNA, a direct product of the activated IRE1*α* ER stress receptor and a widely used indicator of short-term ER stress. It is, therefore, plausible that the mutant SCD1 proteins might induce at least a mild ER stress in the long run in vivo.

Nevertheless, it is important to note that, in addition to in silico analyses and in vitro or in vivo effect studies, the impact of environmental factors fine-tuned by genetic mutations can only be fully understood when they are considered in combination. The role of gene–environment interactions is particularly pronounced in lipid metabolism-related conditions [8,57,58,59], as evidenced by the influence of natural genetic variants on *SCD1* promoter activity [24] or protein stability [23], among other factors, in a FA-dependent manner.

In summary, the results of our study allowed us to establish a severity ranking of the effects of missense mutations on SCD1. Our findings indicated that p.R253AfsTer7 was the most damaging in terms of structure, activity, quantity, and the ER stress caused. The effect of the p.H125P mutation was also pronounced, as it was characterized by a significantly lower protein amount and reduced desaturation activity, likely due to the structural change also predicted in silico. In addition, the *XBP1* branch of the ER stress also seems to be activated in the presence of these mutations. The p.A333T variant had only a moderately deleterious effect on SCD1, as reduced enzyme activity and expression were observed in response to this missense mutation, although neither was a major change in the enzyme structurally predicted, nor did any signs of ER stress emerge. In contrast, the p.M224L variant did not exhibit any adverse effects on SCD1 in any of the parameters tested. It even resulted in an increase in the amount of the protein.

## 5. Conclusions

The development of lipid metabolism-related conditions can be attributed to genetic predisposition, diet, and lifestyle. The individual-specific level of SCD1, and, thus, the amount of de novo synthesized cis UFAs, is determined by the interaction between environmental influences and genetic factors. Therefore, in addition to the identification of natural genetic variations, there is a pressing need for a comprehensive functional characterization of these variations, as this may facilitate more personalized prevention and treatment of lipid metabolism-related adverse conditions.

## Data Availability

All data are available in the main text or the Supplementary Materials. Research materials used in the article can be requested from the authors. This study includes no data deposited in external repositories.

## Supplementary Materials

The following supporting information can be downloaded at https://www.mdpi.com/article/10.3390/nu16193259/s1, Figure S1: Predicted effects of the (A) p.H125P, (B) p.M224L, (C) p.A333T, and (D) p.R253AfsTer7 variants on the spatial structure of SCD1; Figure S2: Desaturation index upon overexpression of p.H125P, p.M224L, p.A333T, and p.R253AfsTer7 SCD1 variants; Table S1: Default and rescaled impact scores generated by the ten prediction programs for the SCD1 variants; Table S2: Sequence and annealing temperature of cloning and mutagenic primers; Table S3: Protein concentration and GC-MS raw data for HEK293T cells expressing SCD1 mutants.
