# Supplementary material for: Severity Ranking of Missense and Frameshift Genetic Variants in SCD1 by In Silico and In Vitro Functional Analysis
Source: Nutrients. 2024 Sep 26;16(19):3259. doi: 10.3390/nu16193259 (PMC11478377; doi:10.3390/nu16193259)

SUPPLEMENTARY MATERIALS

for

Severity ranking of missense and frameshift genetic variants in SCD1 by *in silico* and *in vitro* functional analysis

Hanna K. Susán[1], Gabriella Orosz[1], Veronika Zámbó[1], Miklós Csala[1] and Éva Kereszturi[1,*]

[1] Semmelweis University, Department of Molecular Biology, Budapest, Hungary;

hanna.krisztina@gmail.com (H.K.S.); orosz.gabriella@phd.semmelweis.hu (G.O.);

zambo.veronika@semmelweis.hu (V.Z.); csala.miklos@semmelweis.hu (M.C.);

kereszturi.eva@semmelweis.hu (É.K.)

[*] Correspondence: kereszturi.eva@semmelweis.hu (É.K.)

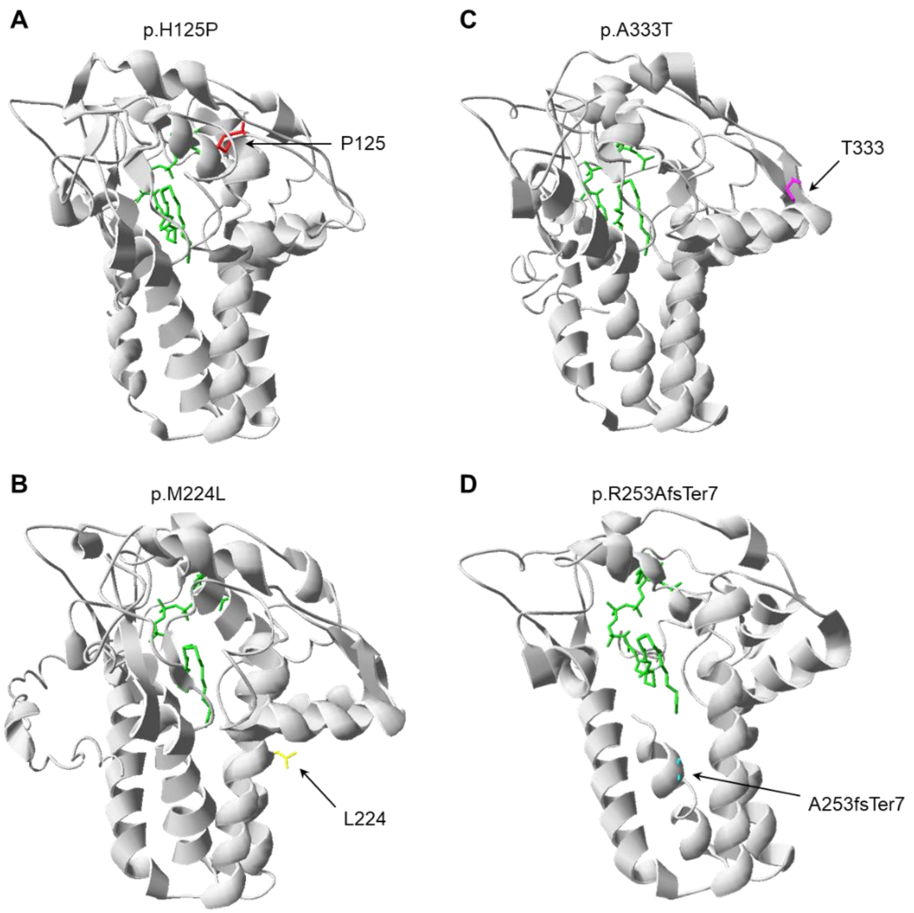

Supplementary Figure S1. Predicted effects of the (***A***) p.H125P, (***B***) p.M224L, (***C***) p.A333T and (***D***) p.R253AfsTer7 variants on the spatial structure of SCD1. 3D structures of the SCD1 variants were predicted by I-TASSER. Images were rendered with DeepView/Swiss-Pdb Viewer version 4.0.2. The affected amino acids P125, L224, T333, A253fsTer7, and stearoyl-CoA are indicated in red, yellow, pink, light blue, and green, respectively.

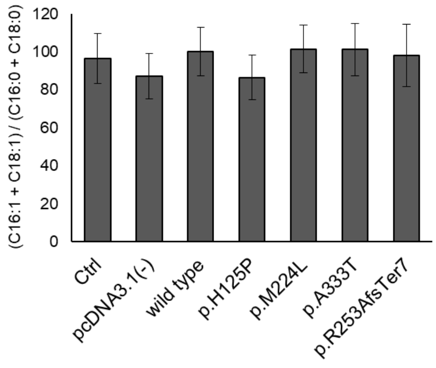

Supplementary Figure S2. Desaturation index upon overexpression of p.H125P, p.M224L, p.A333T and p.R253AfsTer7 SCD1 variants. Transiently transfected HEK293T cells were harvested and the amount of major saturated (C16:0, C18:0) and mono-unsaturated (C16:1c9; C18:1c9) FAs was measured by GC-MS after sample preparation as described in the *Materials and Methods* section. The ratio of unsaturated:saturated FAs was then calculated. Data were normalized to the total protein content of the samples and are presented as mean values ± S.D.; n = 3. The desaturation index of the wild-type SCD1 enzyme was considered to be 100%. Statistical analysis was performed using the Tukey–Kramer multiple comparisons test. Ctrl: control.

Supplementary Table S1. Default and rescaled impact scores generated by the ten prediction programs for the SCD1 variants. The boundaries of the deleterious and beneficial ranges are indicated in red and green, respectively. ndr: no defined range

			**p.H125P**		**p.M224L**		**p.A333T**		**p.R253AfsTer7**	
			impact score		***impact score***		***impact score***		***impact score***	
**Tool**	**Range**	**Treshold**	***original***	***100% scale***	***original***	***100% scale***	***original***	***100% scale***	***original***	***100% scale***
SIFT	0 – 1	0.05	0	100	1	0	0	100	–	–
PolyPhen-2	0 – 1	0.5	0.999	100	0	0	0.793	79	–	–
CADD	0 – 100	15	31	31	0	0	25	25	–	–
REVEL	0 – 1	0.5	0.977	98	0.060	6	0.537	54	–	–
MetaLR	0 – 1	0.5	0.983	98	0.003	0	0.526	53	–	–
MutationAssessor	0 – 1	0.65	0.999	100	0	0	0.977	98	–	–
Provean	ndr	-2.5	-9.088	3	1.260	0	-3.388	1	-371.892	100
MutPred2	0 – 1	0.5	0.940	94	0.093	9	0.666	67	–	–
MutationTaster	0 – 100	50	74 / 26	74	7 / 93	7	65 / 35	65	93 / 7	93
M-CAP	0 – 0.1	0.025	0.809	81	0.004	0	0.158	16	–	–

Supplementary Table S2. Sequence and annealing temperature of cloning and mutagenic primers. Restriction endonuclease recognition sites are highlighted in italic bold capital letters. Glu-Glu-tag and nucleotide exchanges are marked in bold lowercase letters.

Primer name	Sequence 5' → 3'	Tm (°C)
SCD1_RNS-S-XhoI	AAA TTT ***CTC GAG*** CTC AGC CCC CTG GAA AGT GAT	54
SCD1_RNS-AS-HindIII	AAA TTT ***AAG CTT*** GGA ACC TGA GGG ACC CCA AAC	48
SCD1_GluGlu-S	**gaa tat atg cct atg gaa** TGA GTT TGG GGT CCC TCA GGT TCC	61
SCD1_GluGlu-AS	**ttc cat agg cat ata ttc** GCC ACT CTT GTA GTT TCC ATC TCC	57
SCD1_H125P-S	CTG TGG AGC C**c**C CGC TCT TAC	60
SCD1_H125P-AS	GTA AGA GCG G**g**G GCT CCA CAG	60
SCD1_M224L-S	TGG CTT GCT G**c**T GAT GTG CTT	54
SCD1_M224L-AS	AAG CAC ATC A**g**C AGC AAG CCA	54
SCD1_A333T-S	CCT CGG TCT G**a**C CTA TGA CCG	58
SCD1_A333T-AS	CGG TCA TAG G**t**C AGA CCG AGG	58
SCD1_R253AfsTer7-S	TTG CCA CTT T_T TGC GAT ATG	48
SCD1_R253AfsTer7-AS	CAT ATC GCA A_A AAG TGG CAA	48

Supplementary Table S3. Protein concentration and GC-MS raw data for HEK293T cells expressing SCD1 mutants.

Sample ID	Protein conc (µg / mL	Sample-Typ	IS1 [nmol]	IS2 [nmol]	FA10:0 Conc	FA10:0 RT	FA10:0 Area	FA12:0 Conc	FA12:0 RT	FA12:0 Area	FA13:0 Conc
**FAME 1_Control A**	703.75	0:Unknown	4.38	2.94	Not Identified			0.02	3.71	4237.00	1.04
**FAME 1_pcDNA3.1- A**	748.93	0:Unknown	4.38	2.94	Not Identified			0.02	3.71	3865.00	1.07
**FAME 1_SCD1 A**	522.29	0:Unknown	4.38	2.94	Not Identified			0.02	3.71	4671.00	1.02
**FAME 1_H125P A**	691.185	0:Unknown	4.38	2.94	Not Identified			0.02	3.71	4156.00	1.04
**FAME 1_M224L A**	579.175	0:Unknown	4.38	2.94	Not Identified			0.02	3.71	4254.00	1.01
**FAME 1_A333T A**	742.25	0:Unknown	4.38	2.94	Not Identified			0.02	3.71	4869.00	1.01
**FAME 1_Tins_del A**	748.415	0:Unknown	4.38	2.94	Not Identified			0.02	3.71	4530.00	1.00
**FAME 2_Control A**	720.9	0:Unknown	4.38	2.94	Not Identified			0.01	3.71	3250.00	1.08
**FAME 2_pcDNA3.1- A**	934.385	0:Unknown	4.38	2.94	Not Identified			0.02	3.71	4196.00	1.10
**FAME 2_SCD1 A**	543.15	0:Unknown	4.38	2.94	Not Identified			0.02	3.71	4960.00	1.08
**FAME 2_H125P A**	967.38	0:Unknown	4.38	2.94	Not Identified			0.02	3.71	4600.00	1.09
**FAME 2_M224L A**	514.84	0:Unknown	4.38	2.94	Not Identified			0.02	3.71	3779.00	1.08
**FAME 2_A333T A**	765.85	0:Unknown	4.38	2.94	Not Identified			0.02	3.71	3437.00	1.10
**FAME 2_Tins_del A**	1056.8	0:Unknown	4.38	2.94	Not Identified			0.02	3.71	5834.00	1.04
**FAME 3_Control A**	361.37	0:Unknown	4.38	2.94	Not Identified			0.01	3.71	2747.00	1.12
**FAME 3_pcDNA3.1- A**	384.245	0:Unknown	4.38	2.94	Not Identified			0.02	3.71	4269.00	1.16
**FAME 3_SCD1 A**	471.83	0:Unknown	4.38	2.94	Not Identified			0.02	3.71	3967.00	1.13
**FAME 3_H125P A**	392.725	0:Unknown	4.38	2.94	Not Identified			0.02	3.71	8733.00	1.01
**FAME 3_M224L A**	307.045	0:Unknown	4.38	2.94	Not Identified			0.02	3.71	4072.00	1.14
**FAME 3_A333T A**	661.385	0:Unknown	4.38	2.94	Not Identified			0.02	3.71	5498.00	1.11
**FAME 3_Tins_del A**	405.145	0:Unknown	4.38	2.94	Not Identified			0.03	3.71	9544.00	1.05

Supplementary Table S3. Continued.

Sample ID	FA13:0 RT	FA13:0 Area	FA14:0 Conc	FA14:0 RT	FA14:0 Area	FA14:1-c9 Conc	FA14:1-c9 RT	FA14:1-c9 Area	FA15:0 Conc	FA15:0 RT	FA15:0 Area
**FAME 1_Control A**	4.01	304226.00	0.33	4.38	99486.00	Not Identified			0.08	4.83	24084.00
**FAME 1_pcDNA3.1- A**	4.01	227301.00	0.40	4.38	89424.00	Not Identified			0.07	4.83	15518.00
**FAME 1_SCD1 A**	4.01	308429.00	0.38	4.38	119193.00	Not Identified			0.06	4.83	20341.00
**FAME 1_H125P A**	4.01	260833.00	0.39	4.38	100203.00	Not Identified			0.06	4.83	17477.00
**FAME 1_M224L A**	4.01	278367.00	0.39	4.38	110415.00	Not Identified			0.06	4.83	19221.00
**FAME 1_A333T A**	4.01	316767.00	0.42	4.38	136973.00	Not Identified			0.07	4.83	23923.00
**FAME 1_Tins_del A**	4.01	267071.00	0.53	4.38	145114.00	Not Identified			0.09	4.83	25312.00
**FAME 2_Control A**	4.01	279620.00	0.20	4.38	53003.00	Not Identified			0.05	4.83	15278.00
**FAME 2_pcDNA3.1- A**	4.02	281015.00	0.27	4.39	71337.00	Not Identified			0.04	4.84	10526.00
**FAME 2_SCD1 A**	4.02	307050.00	0.29	4.39	84943.00	Not Identified			0.04	4.84	11958.00
**FAME 2_H125P A**	4.01	322893.00	0.29	4.39	89769.00	Not Identified			0.04	4.84	13306.00
**FAME 2_M224L A**	4.01	272113.00	0.28	4.38	72776.00	Not Identified			0.04	4.83	9962.00
**FAME 2_A333T A**	4.01	239161.00	0.29	4.38	64723.00	Not Identified			0.04	4.83	8785.00
**FAME 2_Tins_del A**	4.01	405049.00	0.28	4.38	113256.00	Not Identified			0.04	4.83	15687.00
**FAME 3_Control A**	4.01	247315.00	0.26	4.38	59768.00	Not Identified			0.07	4.83	15895.00
**FAME 3_pcDNA3.1- A**	4.01	272457.00	0.35	4.38	84877.00	Not Identified			0.06	4.83	15106.00
**FAME 3_SCD1 A**	4.01	246308.00	0.36	4.38	80146.00	Not Identified			0.06	4.83	14067.00
**FAME 3_H125P A**	4.01	595310.00	0.29	4.38	179014.00	Not Identified			0.05	4.83	31538.00
**FAME 3_M224L A**	4.01	247390.00	0.32	4.38	73172.00	Not Identified			0.05	4.83	12864.00
**FAME 3_A333T A**	4.01	283230.00	0.34	4.38	88916.00	Not Identified			0.06	4.83	15479.00
**FAME 3_Tins_del A**	4.01	392399.00	0.32	4.38	122765.00	Not Identified			0.05	4.83	20603.00

Supplementary Table S3. Continued.

Sample ID	DM16:0 Conc	DM16:0 RT	DM16:0 Area	FA16:0iso Conc	FA16:0iso RT	FA16:0iso Area	DM16:1 Conc	DM16:1 RT	DM16:1 Area	FA16:0 Conc
**FAME 1_Control A**	0.47	4.96	156824.00	0.02	5.10	5546.00	0.26	5.28	85993.00	2.06
**FAME 1_pcDNA3.1- A**	0.58	4.97	139682.00	0.02	5.10	4379.00	0.26	5.28	62020.00	2.39
**FAME 1_SCD1 A**	0.52	4.97	180748.00	0.02	5.10	5953.00	0.25	5.28	85501.00	2.07
**FAME 1_H125P A**	0.58	4.97	164678.00	0.02	5.10	5001.00	0.26	5.28	73312.00	2.25
**FAME 1_M224L A**	0.53	4.97	164745.00	0.02	5.10	5741.00	0.26	5.28	82434.00	2.11
**FAME 1_A333T A**	0.56	4.97	202280.00	0.02	5.10	7378.00	0.32	5.28	116423.00	2.25
**FAME 1_Tins_del A**	0.59	4.97	177765.00	0.03	5.10	8863.00	0.62	5.28	186638.00	2.59
**FAME 2_Control A**	0.60	4.97	177448.00	0.00	5.10	1355.00	0.08	5.28	22642.00	1.87
**FAME 2_pcDNA3.1- A**	0.58	4.97	168211.00	0.01	5.10	1593.00	0.08	5.28	22247.00	2.04
**FAME 2_SCD1 A**	0.57	4.97	184914.00	0.01	5.10	1954.00	0.08	5.28	25290.00	1.99
**FAME 2_H125P A**	0.65	4.97	220943.00	0.01	5.10	2059.00	0.07	5.28	23837.00	2.21
**FAME 2_M224L A**	0.55	4.97	156297.00	0.01	5.10	1636.00	0.07	5.28	21401.00	1.90
**FAME 2_A333T A**	0.56	4.97	139214.00	0.01	5.10	1363.00	0.08	5.28	19283.00	1.96
**FAME 2_Tins_del A**	0.61	4.97	272427.00	0.01	5.10	2499.00	0.07	5.28	30829.00	2.08
**FAME 3_Control A**	0.59	4.96	148200.00	0.01	5.09	1297.00	0.07	5.28	17051.00	2.19
**FAME 3_pcDNA3.1- A**	0.80	4.96	212459.00	0.01	5.09	2045.00	0.07	5.28	19801.00	2.55
**FAME 3_SCD1 A**	0.77	4.96	189667.00	0.01	5.09	2070.00	0.07	5.27	17470.00	2.39
**FAME 3_H125P A**	0.69	4.96	465495.00	0.01	5.10	4438.00	0.06	5.28	42278.00	2.14
**FAME 3_M224L A**	0.71	4.96	175152.00	0.01	5.09	1838.00	0.07	5.28	16709.00	2.18
**FAME 3_A333T A**	0.74	4.96	215706.00	0.01	5.09	2254.00	0.07	5.28	19969.00	2.28
**FAME 3_Tins_del A**	0.74	4.96	314313.00	0.01	5.09	2786.00	0.06	5.28	27499.00	2.32

Supplementary Table S3. Continued.

Sample ID	FA16:0 RT	FA16:0 Area	FA16:1-c9 Conc	FA16:1-c9 RT	FA16:1-c9 Area	FA16:0-4Me Conc	FA16:0-4Me RT	FA16:0-4Me Area	FA17:0 Conc	FA17:0 RT	FA17:0 Area
**FAME 1_Control A**	5.37	687130.00	0.57	5.63	71627.00	Not Identified			0.05	5.99	14437.00
**FAME 1_pcDNA3.1- A**	5.37	580398.00	0.62	5.63	56965.00	Not Identified			0.05	5.99	10093.00
**FAME 1_SCD1 A**	5.37	716630.00	0.82	5.63	106328.00	Not Identified			0.04	5.99	12295.00
**FAME 1_H125P A**	5.37	640797.00	0.59	5.63	63193.00	Not Identified			0.05	5.99	11617.00
**FAME 1_M224L A**	5.37	657663.00	0.80	5.63	94034.00	Not Identified			0.04	5.99	11597.00
**FAME 1_A333T A**	5.38	804593.00	0.81	5.63	108841.00	Not Identified			0.05	5.99	14458.00
**FAME 1_Tins_del A**	5.38	786304.00	0.75	5.63	85483.00	Not Identified			0.06	5.99	15364.00
**FAME 2_Control A**	5.37	550041.00	0.37	5.63	41533.00	Not Identified			0.04	5.99	9333.00
**FAME 2_pcDNA3.1- A**	5.38	594220.00	0.40	5.63	43855.00	Not Identified			0.03	5.99	7729.00
**FAME 2_SCD1 A**	5.38	645896.00	0.62	5.64	75456.00	Not Identified			0.03	5.99	7713.00
**FAME 2_H125P A**	5.38	745608.00	0.43	5.63	54185.00	Not Identified			0.03	5.99	9833.00
**FAME 2_M224L A**	5.37	546022.00	0.62	5.63	67011.00	Not Identified			0.02	5.99	6213.00
**FAME 2_A333T A**	5.37	487023.00	0.58	5.63	53769.00	Not Identified			0.03	5.99	5662.00
**FAME 2_Tins_del A**	5.38	923612.00	0.41	5.63	68587.00	Not Identified			0.03	5.99	11336.00
**FAME 3_Control A**	5.37	552216.00	0.44	5.63	41336.00	Not Identified			0.04	5.99	9561.00
**FAME 3_pcDNA3.1- A**	5.37	681510.00	0.46	5.63	46437.00	Not Identified			0.05	5.99	11052.00
**FAME 3_SCD1 A**	5.37	592165.00	0.81	5.63	75261.00	Not Identified			0.04	5.98	9031.00
**FAME 3_H125P A**	5.37	1441516.00	0.41	5.63	104743.00	Not Identified			0.04	5.99	24138.00
**FAME 3_M224L A**	5.37	540601.00	0.71	5.63	66719.00	Not Identified			0.04	5.99	8055.00
**FAME 3_A333T A**	5.37	660180.00	0.71	5.63	77972.00	Not Identified			0.04	5.99	10006.00
**FAME 3_Tins_del A**	5.37	981651.00	0.45	5.63	71282.00	Not Identified			0.04	5.99	15467.00

Supplementary Table S3. Continued.

Sample ID	DM18:1 Conc	DM18:1 RT	DM18:1 Area	FA18:0 Conc	FA18:0 RT	FA18:0 Area	FA18:1-c9 (n-9) Conc	FA18:1-c9 (n-9) RT	FA18:1-c9 (n-9) Area	FA18:1-c11 (n-7) Conc
**FAME 1_Control A**	0.11	6.14	38336.00	0.62	6.69	217506.00	4.61	6.97	594882.00	0.41
**FAME 1_pcDNA3.1- A**	0.14	6.14	35084.00	0.79	6.69	198930.00	4.94	6.97	463266.00	0.47
**FAME 1_SCD1 A**	0.12	6.15	42521.00	0.67	6.69	241306.00	4.56	6.97	608071.00	0.58
**FAME 1_H125P A**	0.14	6.15	41211.00	0.83	6.69	247350.00	4.77	6.97	523541.00	0.45
**FAME 1_M224L A**	0.12	6.15	39354.00	0.69	6.69	223020.00	4.70	6.97	566085.00	0.58
**FAME 1_A333T A**	0.13	6.15	49116.00	0.68	6.69	252606.00	5.12	6.98	708139.00	0.60
**FAME 1_Tins_del A**	0.15	6.15	46094.00	0.79	6.70	250153.00	6.50	6.98	760797.00	0.47
**FAME 2_Control A**	0.14	6.15	41809.00	0.69	6.69	211209.00	1.87	6.96	212864.00	0.45
**FAME 2_pcDNA3.1- A**	0.14	6.15	42888.00	0.79	6.69	240861.00	1.89	6.96	212804.00	0.43
**FAME 2_SCD1 A**	0.13	6.15	43334.00	0.72	6.70	242904.00	1.90	6.96	237753.00	0.60
**FAME 2_H125P A**	0.16	6.15	55892.00	0.83	6.70	293729.00	2.04	6.97	265638.00	0.48
**FAME 2_M224L A**	0.12	6.15	36768.00	0.63	6.69	187015.00	1.84	6.96	203720.00	0.60
**FAME 2_A333T A**	0.13	6.15	33471.00	0.66	6.69	171654.00	1.90	6.96	181964.00	0.60
**FAME 2_Tins_del A**	0.15	6.15	70385.00	0.75	6.69	346875.00	1.91	6.97	327689.00	0.45
**FAME 3_Control A**	0.14	6.14	36688.00	0.81	6.69	213171.00	1.85	6.96	179291.00	0.42
**FAME 3_pcDNA3.1- A**	0.20	6.14	55025.00	1.22	6.69	337717.00	2.05	6.96	210984.00	0.48
**FAME 3_SCD1 A**	0.18	6.14	45245.00	1.13	6.69	291916.00	2.07	6.96	197633.00	0.72
**FAME 3_H125P A**	0.18	6.14	123030.00	1.15	6.70	806340.00	1.76	6.97	457382.00	0.43
**FAME 3_M224L A**	0.16	6.14	41991.00	0.91	6.69	234058.00	1.81	6.96	172839.00	0.63
**FAME 3_A333T A**	0.17	6.14	51723.00	1.10	6.69	333222.00	1.93	6.96	215628.00	0.68
**FAME 3_Tins_del A**	0.19	6.14	82511.00	1.11	6.69	488901.00	1.83	6.96	299630.00	0.47

Supplementary Table S3. Continued.

Sample ID	FA18:1-c11 (n-7) RT	FA18:1-c11 (n-7) Area	FA18:2-c9,c12 (n-6) Conc	FA18:2-c9,c12 (n-6) RT	FA18:2-c9,c12 (n-6) Area	FA18:3-c6,c9,c12 (n-6) Conc	FA18:3-c6,c9,c12 (n-6) RT	FA18:3-c6,c9,c12 (n-6) Area	FA18:3-c9,c12,c15 (n-3) Conc	FA18:3-c9,c12,c15 (n-3) RT	FA18:3-c9,c12,c15 (n-3) Area
**FAME 1_Control A**	7.03	60282.00	0.07	7.45	10341.00	Not Identified			Not Identified		
**FAME 1_pcDNA3.1- A**	7.03	49456.00	0.08	7.45	9074.00	Not Identified			Not Identified		
**FAME 1_SCD1 A**	7.03	87342.00	0.07	7.45	11762.00	Not Identified			Not Identified		
**FAME 1_H125P A**	7.03	56263.00	0.08	7.45	10421.00	Not Identified			Not Identified		
**FAME 1_M224L A**	7.03	79571.00	0.08	7.45	10904.00	Not Identified			Not Identified		
**FAME 1_A333T A**	7.03	93704.00	0.08	7.45	13336.00	Not Identified			Not Identified		
**FAME 1_Tins_del A**	7.03	62652.00	0.10	7.45	13249.00	Not Identified			Not Identified		
**FAME 2_Control A**	7.03	57776.00	0.03	7.45	4576.00	Not Identified			Not Identified		
**FAME 2_pcDNA3.1- A**	7.03	54986.00	0.04	7.45	5084.00	Not Identified			Not Identified		
**FAME 2_SCD1 A**	7.03	85122.00	0.04	7.45	5243.00	Not Identified			Not Identified		
**FAME 2_H125P A**	7.03	70354.00	0.04	7.45	6538.00	Not Identified			Not Identified		
**FAME 2_M224L A**	7.03	75528.00	0.03	7.45	4341.00	Not Identified			Not Identified		
**FAME 2_A333T A**	7.03	65343.00	0.04	7.45	4029.00	Not Identified			Not Identified		
**FAME 2_Tins_del A**	7.03	86917.00	0.04	7.45	7817.00	Not Identified			Not Identified		
**FAME 3_Control A**	7.02	46144.00	0.04	7.44	4856.00	Not Identified			Not Identified		
**FAME 3_pcDNA3.1- A**	7.02	55632.00	0.06	7.44	7744.00	Not Identified			Not Identified		
**FAME 3_SCD1 A**	7.02	77803.00	0.06	7.44	7029.00	Not Identified			Not Identified		
**FAME 3_H125P A**	7.03	127327.00	0.06	7.45	17546.00	Not Identified			Not Identified		
**FAME 3_M224L A**	7.02	68574.00	0.06	7.44	6377.00	Not Identified			Not Identified		
**FAME 3_A333T A**	7.02	86235.00	0.06	7.44	7900.00	Not Identified			Not Identified		
**FAME 3_Tins_del A**	7.02	87290.00	0.06	7.44	11109.00	Not Identified			Not Identified		

Supplementary Table S3. Continued.

Sample ID	FA20:0 Conc	FA20:0 RT	FA20:0 Area	FA20:1-c11 (n-9) Conc	FA20:1-c11 (n-9) RT	FA20:1-c11 (n-9) Area	FA21:0iso Conc	FA21:0iso RT	FA21:0iso Area
**FAME 1_Control A**		Not Identified		0.03	8.55	4320.00	1.00	8.68	208832.00
**FAME 1_pcDNA3.1- A**		Not Identified		0.04	8.55	3746.00	1.00	8.68	151812.00
**FAME 1_SCD1 A**		Not Identified		0.04	8.55	5210.00	1.00	8.68	216129.00
**FAME 1_H125P A**		Not Identified		0.03	8.55	4208.00	1.00	8.68	177868.00
**FAME 1_M224L A**		Not Identified		0.04	8.55	4764.00	1.00	8.68	195100.00
**FAME 1_A333T A**		Not Identified		0.04	8.55	6151.00	1.00	8.68	223918.00
**FAME 1_Tins_del A**		Not Identified		0.04	8.56	5535.00	1.00	8.68	189591.00
**FAME 2_Control A**	0.01	8.25	3096.00	0.03	8.55	3906.00	1.00	8.68	184145.00
**FAME 2_pcDNA3.1- A**	0.02	8.26	4420.00	0.03	8.56	3960.00	1.00	8.68	182370.00
**FAME 2_SCD1 A**	0.01	8.26	4152.00	0.03	8.56	3822.00	1.00	8.68	202850.00
**FAME 2_H125P A**	0.02	8.26	5488.00	0.03	8.56	4979.00	1.00	8.68	211113.00
**FAME 2_M224L A**	0.01	8.26	3605.00	0.03	8.56	3319.00	1.00	8.68	179184.00
**FAME 2_A333T A**	0.01	8.26	3057.00	0.03	8.56	2931.00	1.00	8.68	155134.00
**FAME 2_Tins_del A**	0.02	8.25	6823.00	0.03	8.56	4878.00	1.00	8.68	277557.00
**FAME 3_Control A**	0.01	8.25	3196.00	0.03	8.55	2728.00	1.00	8.68	157251.00
**FAME 3_pcDNA3.1- A**	0.02	8.25	4384.00	0.03	8.55	3388.00	1.00	8.68	166730.00
**FAME 3_SCD1 A**	0.01	8.25	3624.00	0.03	8.55	3242.00	1.00	8.68	154736.00
**FAME 3_H125P A**	0.02	8.25	10473.00	0.03	8.55	8301.00	1.00	8.69	420692.00
**FAME 3_M224L A**	0.01	8.25	3243.00	0.03	8.55	3258.00	1.00	8.68	154894.00
**FAME 3_A333T A**	0.01	8.25	3940.00	0.03	8.55	3722.00	1.00	8.68	181062.00
**FAME 3_Tins_del A**	0.02	8.25	6798.00	0.03	8.55	5389.00	1.00	8.68	264835.00

Supplementary Table S3. Continued.

Sample ID	FA20:2-11c,14c Conc	FA20:2-11c,14c RT	FA20:2-11c,14c Area	FA20:3-c8,c11,c14 (n-6) Conc	FA20:3-c8,c11,c14 (n-6) RT	FA20:3-c8,c11,c14 (n-6) Area	FA20:4-c4,c8,c11,c14 (n-6) Conc	FA20:4-c4,c8,c11,c14 (n-6) RT	FA20:4-c4,c8,c11,c14 (n-6) Area	FA20:3-c11,c14,c17 (n-3) Conc	FA20:3-c11,c14,c17 (n-3) RT	FA20:3-c11,c14,c17 (n-3) Area	FA22:0 Conc
**FAME 1_Control A**	Not Identified			0.03	9.45	3221.00	0.09	9.69	10558.00	Not Identified			0.02
**FAME 1_pcDNA3.1- A**	Not Identified			0.03	9.46	2915.00	0.13	9.69	10804.00	Not Identified			0.02
**FAME 1_SCD1 A**	Not Identified			0.03	9.46	3853.00	0.12	9.69	14501.00	Not Identified			0.02
**FAME 1_H125P A**	Not Identified			0.03	9.46	3431.00	0.13	9.69	12664.00	Not Identified			0.02
**FAME 1_M224L A**	Not Identified			0.03	9.46	3451.00	0.12	9.69	13275.00	Not Identified			0.02
**FAME 1_A333T A**	Not Identified			0.03	9.46	4635.00	0.13	9.69	16106.00	Not Identified			0.02
**FAME 1_Tins_del A**	Not Identified			0.03	9.46	3612.00	0.13	9.69	13393.00	Not Identified			0.02
**FAME 2_Control A**	Not Identified			0.02	9.46	2699.00	0.09	9.69	9228.00	Not Identified			0.02
**FAME 2_pcDNA3.1- A**	Not Identified			0.03	9.46	2806.00	0.10	9.69	9676.00	Not Identified			0.02
**FAME 2_SCD1 A**	Not Identified			0.02	9.46	2972.00	0.09	9.69	10690.00	Not Identified			0.02
**FAME 2_H125P A**	Not Identified			0.03	9.46	3778.00	0.11	9.69	13260.00	Not Identified			0.02
**FAME 2_M224L A**	Not Identified			0.02	9.46	2476.00	0.09	9.69	9066.00	Not Identified			0.02
**FAME 2_A333T A**	Not Identified			0.02	9.45	2306.00	0.10	9.69	8339.00	Not Identified			0.02
**FAME 2_Tins_del A**	Not Identified			0.03	9.46	4499.00	0.10	9.69	15708.00	Not Identified			0.03
**FAME 3_Control A**	Not Identified			0.03	9.45	2819.00	0.11	9.69	9636.00	Not Identified			0.01
**FAME 3_pcDNA3.1- A**	Not Identified			0.04	9.45	4506.00	0.18	9.69	16692.00	Not Identified			0.02
**FAME 3_SCD1 A**	Not Identified			0.04	9.45	4153.00	0.20	9.69	17192.00	Not Identified			0.02
**FAME 3_H125P A**	Not Identified			0.04	9.45	11236.00	0.18	9.69	41611.00	Not Identified			0.02
**FAME 3_M224L A**	Not Identified			0.04	9.45	3855.00	0.18	9.69	15446.00	Not Identified			0.02
**FAME 3_A333T A**	Not Identified			0.04	9.45	4851.00	0.19	9.69	19484.00	Not Identified			0.02
**FAME 3_Tins_del A**	Not Identified			0.04	9.46	6899.00	0.18	9.69	26797.00	Not Identified			0.02

Supplementary Table S3. Continued.

Sample ID	FA22:0 RT	FA22:0 Area	FA20:4-c8,c11,c14,c17 (n-3) Conc	FA20:4-c8,c11,c14,c17 (n-3) RT	FA20:4-c8,c11,c14,c17 (n-3) Area	FA22:1-c13 (n-9) Conc	FA22:1-c13 (n-9) RT	FA22:1-c13 (n-9) Area	FA20:5-c5,c8,c11,c14,c17 Conc	FA20:5-c5,c8,c11,c14,c17 RT	FA20:5-c5,c8,c11,c14,c17 Area
**FAME 1_Control A**	9.93	5139.00	Not Identified			0.06	10.26	6871.00	0.02	10.39	2391.00
**FAME 1_pcDNA3.1- A**	9.93	3806.00	Not Identified			0.06	10.26	5053.00	0.02	10.39	2068.00
**FAME 1_SCD1 A**	9.94	4605.00	Not Identified			0.06	10.26	6967.00	0.02	10.39	2553.00
**FAME 1_H125P A**	9.94	4019.00	Not Identified			0.06	10.26	5845.00	0.02	10.39	2485.00
**FAME 1_M224L A**	9.94	3788.00	Not Identified			0.06	10.26	6598.00	0.02	10.39	2391.00
**FAME 1_A333T A**	9.94	6657.00	Not Identified			0.06	10.26	7491.00	0.02	10.39	2928.00
**FAME 1_Tins_del A**	9.94	5249.00	Not Identified			0.07	10.26	7333.00	0.02	10.39	2802.00
**FAME 2_Control A**	9.93	3652.00	Not Identified			0.03	10.26	2797.00	0.01	10.39	1655.00
**FAME 2_pcDNA3.1- A**	9.94	4953.00	Not Identified			0.03	10.26	3124.00	0.02	10.39	1826.00
**FAME 2_SCD1 A**	9.94	4809.00	Not Identified			0.03	10.26	3545.00	0.01	10.39	1758.00
**FAME 2_H125P A**	9.94	5321.00	Not Identified			0.03	10.25	3560.00	0.02	10.39	2434.00
**FAME 2_M224L A**	9.94	3628.00	Not Identified			0.03	10.26	3006.00	0.01	10.39	1508.00
**FAME 2_A333T A**	9.94	4558.00	Not Identified			0.03	10.26	2691.00	0.01	10.39	1365.00
**FAME 2_Tins_del A**	9.94	9574.00	Not Identified			0.03	10.26	4587.00	0.02	10.39	3015.00
**FAME 3_Control A**	9.93	2720.00	Not Identified			0.03	10.26	2279.00	0.02	10.38	1699.00
**FAME 3_pcDNA3.1- A**	9.93	4410.00	Not Identified			0.03	10.25	2964.00	0.03	10.39	3166.00
**FAME 3_SCD1 A**	9.93	3530.00	Not Identified			0.03	10.25	2893.00	0.03	10.38	2726.00
**FAME 3_H125P A**	9.94	10664.00	Not Identified			0.03	10.26	7999.00	0.03	10.39	7761.00
**FAME 3_M224L A**	9.93	3334.00	Not Identified			0.03	10.26	2552.00	0.02	10.39	2476.00
**FAME 3_A333T A**	9.94	3709.00	Not Identified			0.03	10.26	3380.00	0.03	10.39	3086.00
**FAME 3_Tins_del A**	9.94	7141.00	Not Identified			0.03	10.26	4974.00	0.03	10.39	4781.00

Supplementary Table S3. Continued.

Sample ID	FA23:0 Conc	FA23:0 RT	FA23:0 Area	FA22:4-(7c,10c,13c,16c) Conc	FA22:4-(7c,10c,13c,16c) RT	FA22:4-(7c,10c,13c,16c) Area	FA24:0 Conc	FA24:0 RT	FA24:0 Area	FA24:1-c15 (n-9) Conc	FA24:1-c15 (n-9) RT	FA24:1-c15 (n-9) Area	FA22:5-(7c,10c,13c,16c,19c) Conc	FA22:5-(7c,10c,13c,16c,19c) RT
**FAME 1_Control A**	Not Identified			Not Identified			Not Identified			0.01	11.97	772.00	0.02	12.22
**FAME 1_pcDNA3.1- A**	Not Identified			Not Identified			Not Identified			0.01	11.97	735.00	0.03	12.21
**FAME 1_SCD1 A**	Not Identified			Not Identified			Not Identified			0.01	11.97	1140.00	0.04	12.22
**FAME 1_H125P A**	Not Identified			Not Identified			Not Identified			0.01	11.97	853.00	0.03	12.22
**FAME 1_M224L A**	Not Identified			Not Identified			Not Identified			0.01	11.97	1014.00	0.03	12.22
**FAME 1_A333T A**	Not Identified			Not Identified			Not Identified			0.02	11.97	1265.00	0.04	12.22
**FAME 1_Tins_del A**	Not Identified			Not Identified			Not Identified			0.01	11.97	866.00	0.04	12.22
**FAME 2_Control A**	Not Identified			Not Identified			Not Identified			0.01	11.97	734.00	0.02	12.21
**FAME 2_pcDNA3.1- A**	Not Identified			Not Identified			Not Identified			0.01	11.96	883.00	0.03	12.21
**FAME 2_SCD1 A**	Not Identified			Not Identified			Not Identified			0.02	11.97	1203.00	0.03	12.22
**FAME 2_H125P A**	Not Identified			Not Identified			Not Identified			0.01	11.97	1080.00	0.03	12.21
**FAME 2_M224L A**	Not Identified			Not Identified			Not Identified			0.02	11.97	1174.00	0.02	12.21
**FAME 2_A333T A**	Not Identified			Not Identified			Not Identified			0.02	11.97	863.00	0.03	12.21
**FAME 2_Tins_del A**	Not Identified			Not Identified			Not Identified			0.02	11.97	1578.00	0.03	12.21
**FAME 3_Control A**	Not Identified			Not Identified			Not Identified			0.01	11.97	690.00	0.03	12.21
**FAME 3_pcDNA3.1- A**	Not Identified			Not Identified			Not Identified			0.02	11.97	1464.00	0.06	12.21
**FAME 3_SCD1 A**	Not Identified			Not Identified			Not Identified			0.03	11.97	1589.00	0.06	12.21
**FAME 3_H125P A**	Not Identified			Not Identified			Not Identified			0.02	11.97	3326.00	0.06	12.21
**FAME 3_M224L A**	Not Identified			Not Identified			Not Identified			0.03	11.97	1571.00	0.05	12.21
**FAME 3_A333T A**	Not Identified			Not Identified			Not Identified			0.03	11.97	1750.00	0.06	12.21
**FAME 3_Tins_del A**	Not Identified			Not Identified			Not Identified			0.02	11.97	2370.00	0.06	12.21

Supplementary Table S3. Continued.

Sample ID	FA22:5-(7c,10c,13c,16c,19c) Area	FA22:6-c4,c7,c10,c13,c16,c19 (n-3) Conc	FA22:6-c4,c7,c10,c13,c16,c19 (n-3) RT	FA22:6-c4,c7,c10,c13,c16,c19 (n-3) Area
**FAME 1_Control A**	2533.00	0.04	12.42	5139.00
**FAME 1_pcDNA3.1- A**	2550.00	0.07	12.42	5638.00
**FAME 1_SCD1 A**	3682.00	0.07	12.42	7890.00
**FAME 1_H125P A**	2969.00	0.07	12.42	6908.00
**FAME 1_M224L A**	3097.00	0.06	12.42	6972.00
**FAME 1_A333T A**	4624.00	0.07	12.42	8753.00
**FAME 1_Tins_del A**	3593.00	0.07	12.42	7308.00
**FAME 2_Control A**	1935.00	0.04	12.42	3702.00
**FAME 2_pcDNA3.1- A**	2223.00	0.04	12.42	4168.00
**FAME 2_SCD1 A**	2491.00	0.04	12.42	4586.00
**FAME 2_H125P A**	3002.00	0.05	12.42	5969.00
**FAME 2_M224L A**	2130.00	0.04	12.42	3777.00
**FAME 2_A333T A**	1951.00	0.04	12.42	3624.00
**FAME 2_Tins_del A**	3878.00	0.05	12.42	7454.00
**FAME 3_Control A**	2108.00	0.05	12.42	4440.00
**FAME 3_pcDNA3.1- A**	4791.00	0.11	12.42	10107.00
**FAME 3_SCD1 A**	4575.00	0.11	12.42	9528.00
**FAME 3_H125P A**	12012.00	0.12	12.42	26998.00
**FAME 3_M224L A**	4084.00	0.10	12.42	8654.00
**FAME 3_A333T A**	5391.00	0.11	12.42	11465.00
**FAME 3_Tins_del A**	7435.00	0.11	12.42	16089.00

UNCROPPED PARALLEL BLOT IMAGES

Protein levels of SCD1 missense mutants in transiently transfected HEK293T cells

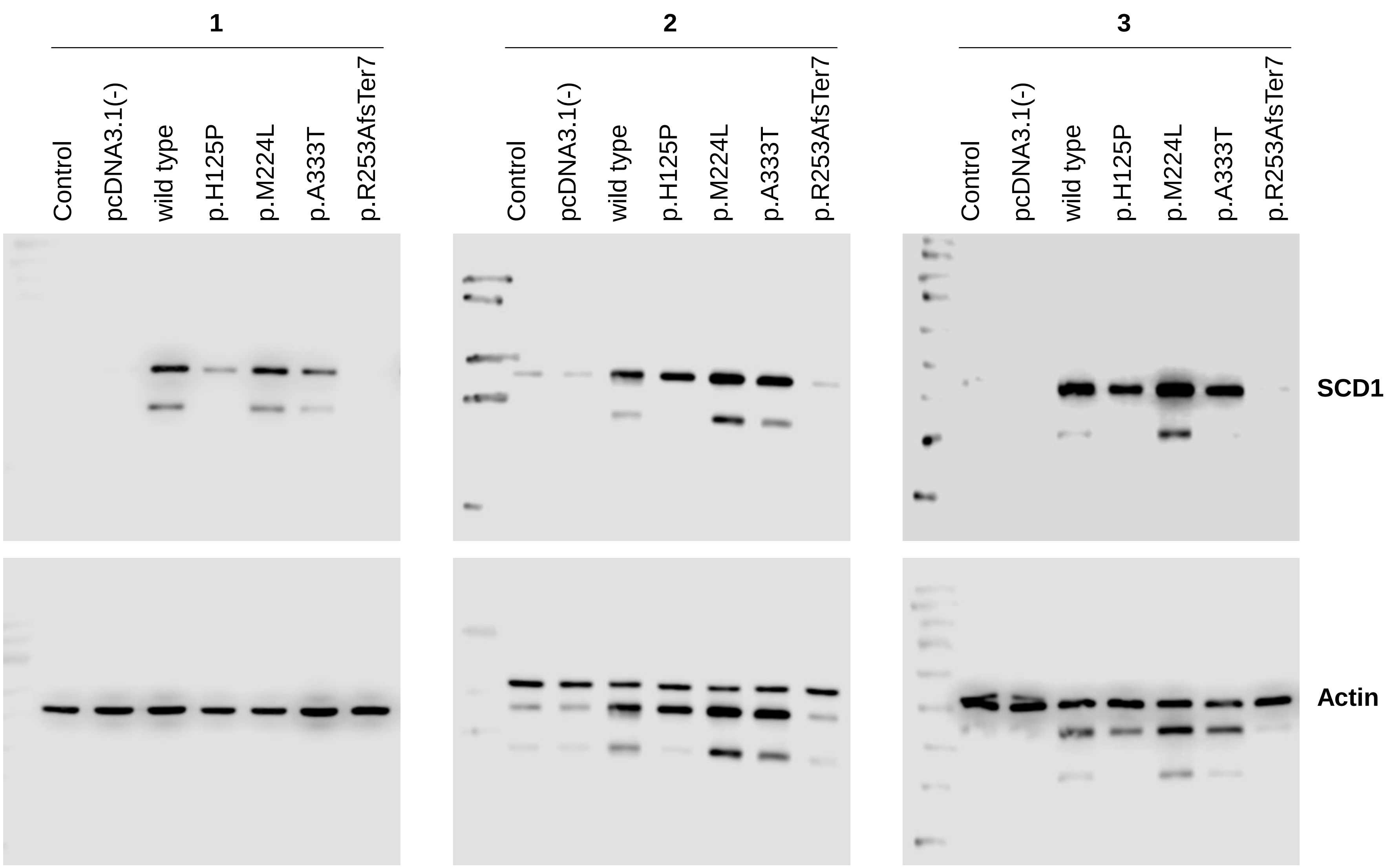

Protein levels of Glu-Glu-tagged SCD1 missense mutants in transiently transfected HEK293T cells

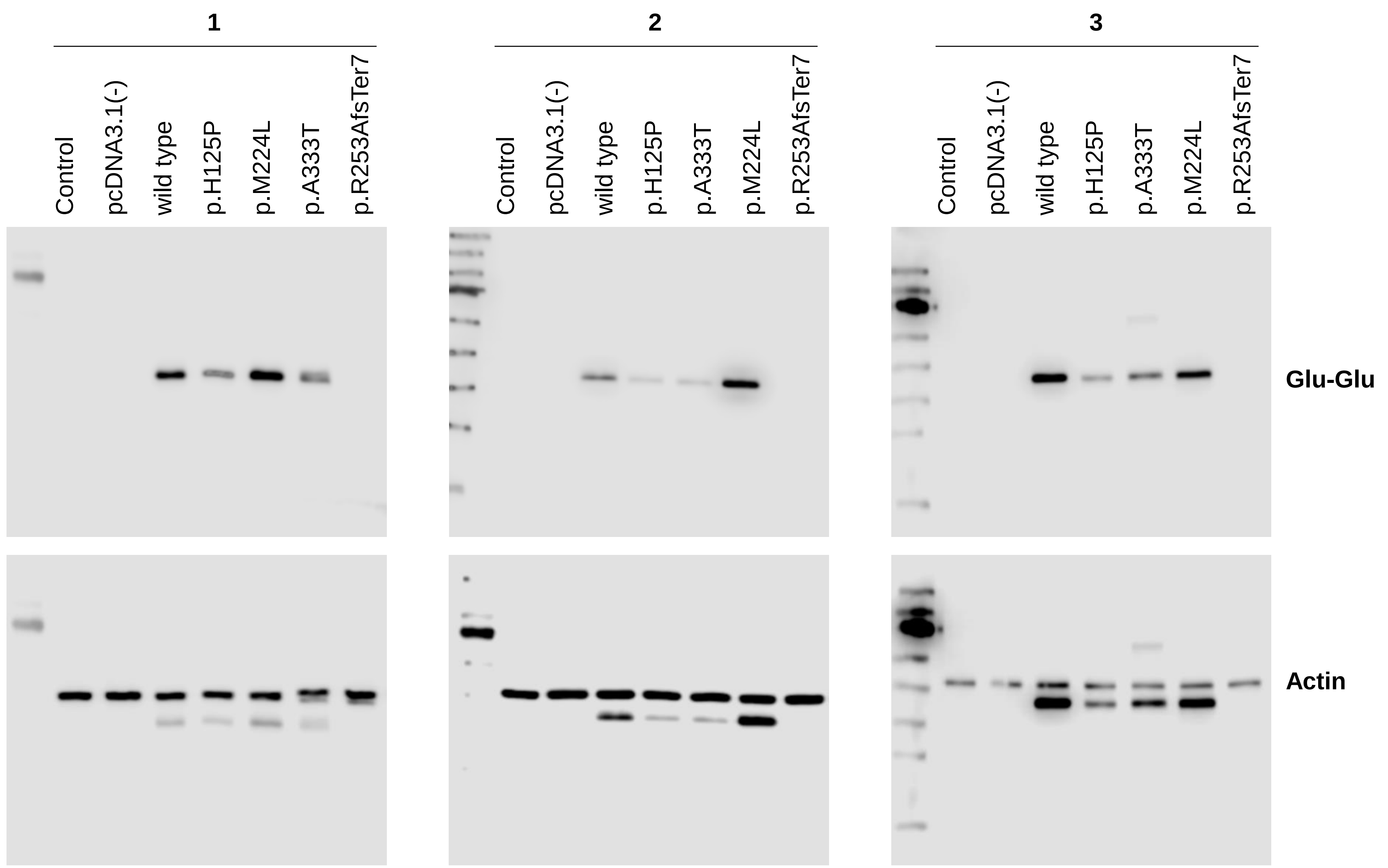

Protein levels of Glu-Glu-tagged SCD1 missense mutants in transiently transfected SK-N-FI cells

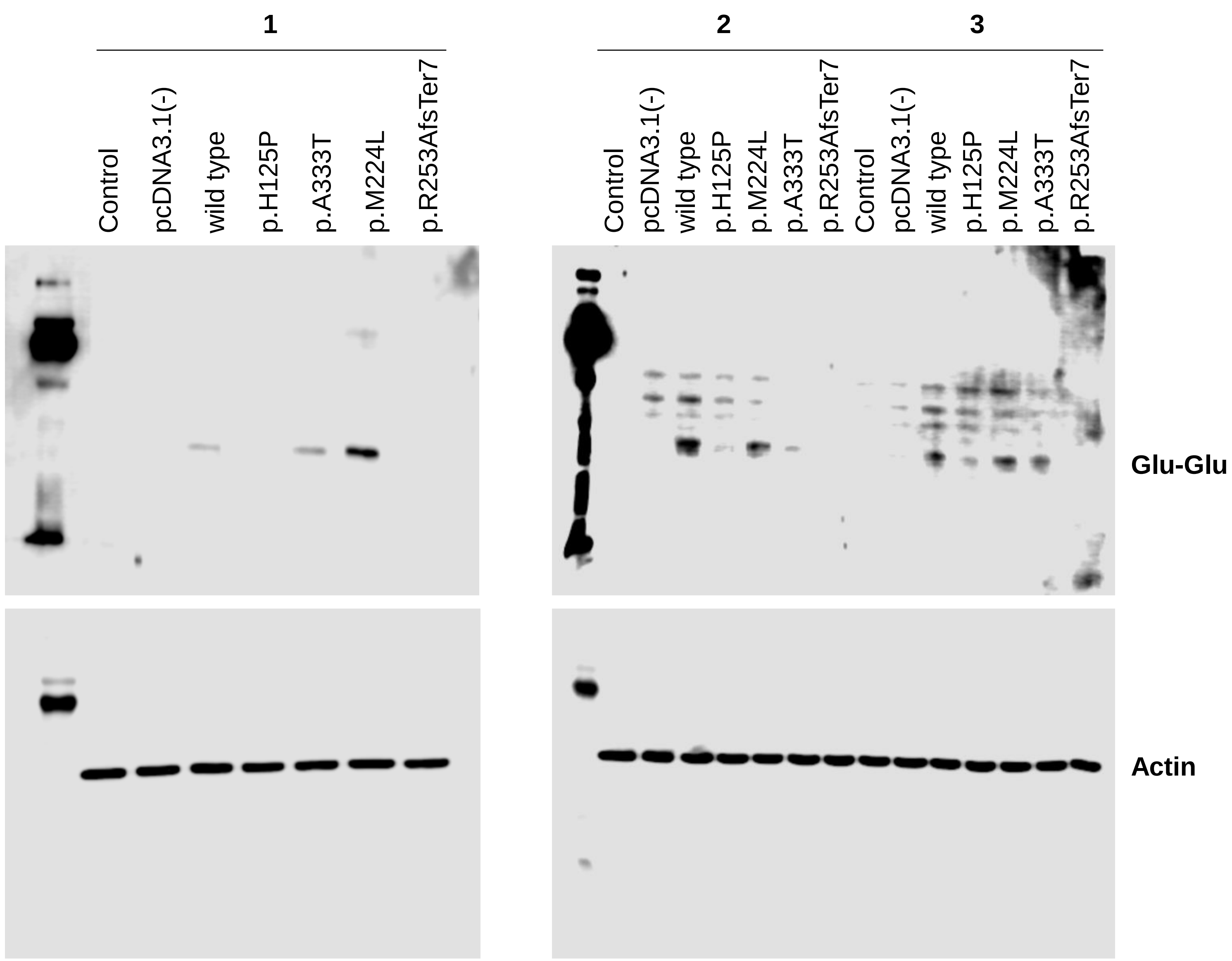

Protein levels of SCD1 missense mutants in HEK293T cells treated with 2 µM MG132 for 24h

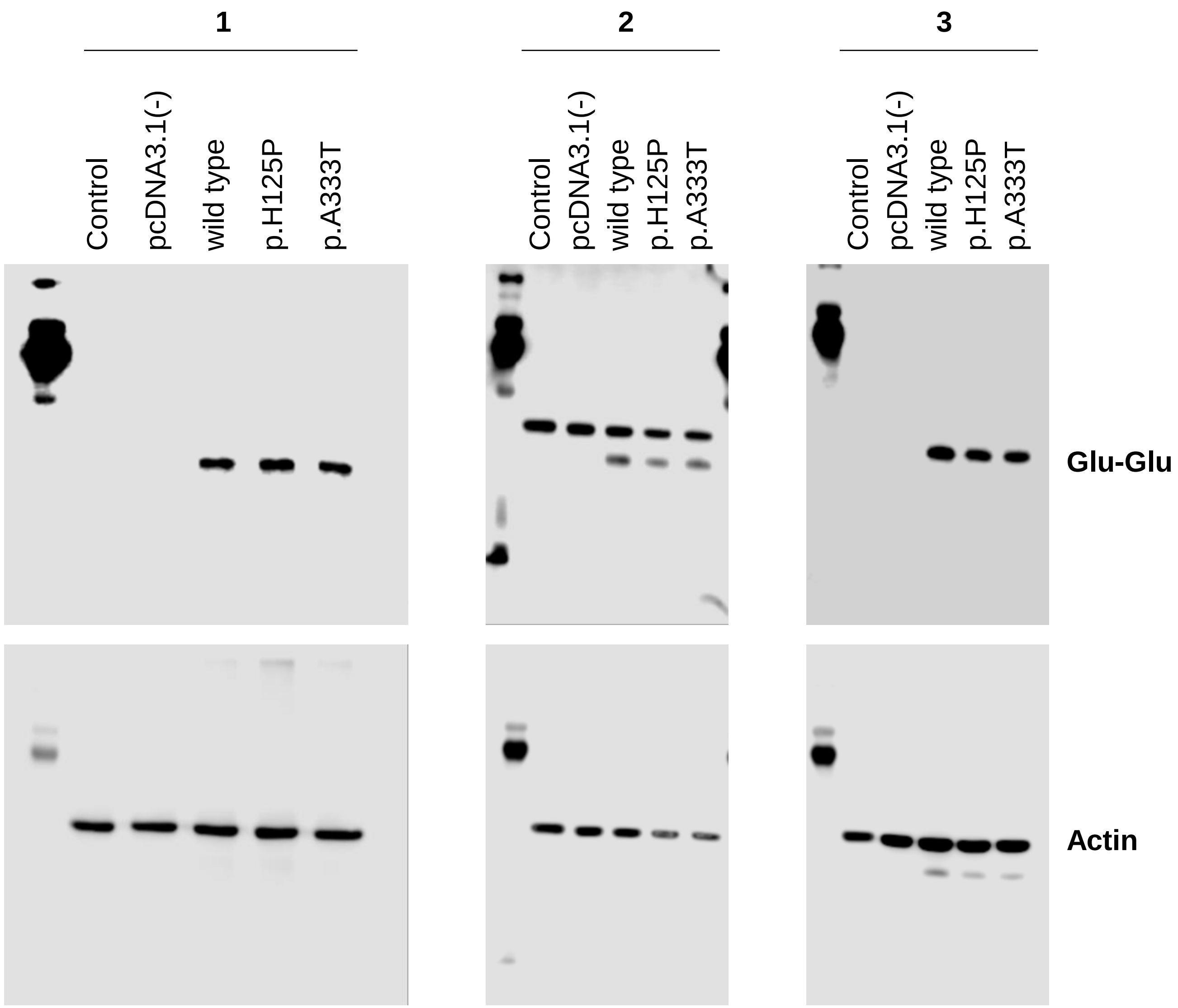

SCD1 protein content of pellet and supernatant from transiently transfected HEK293T cell lysates

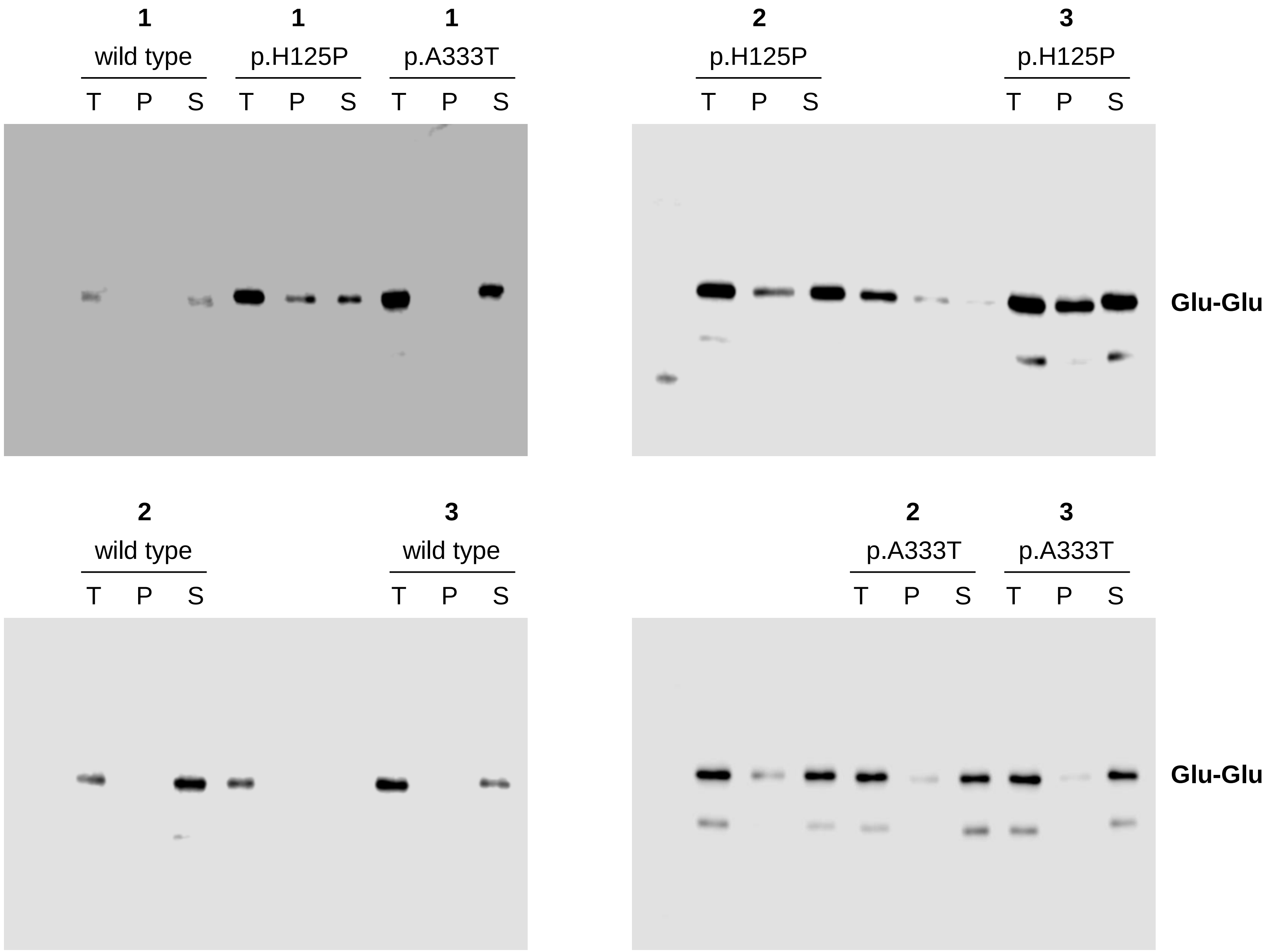

SCD1 protein levels after protease digestion

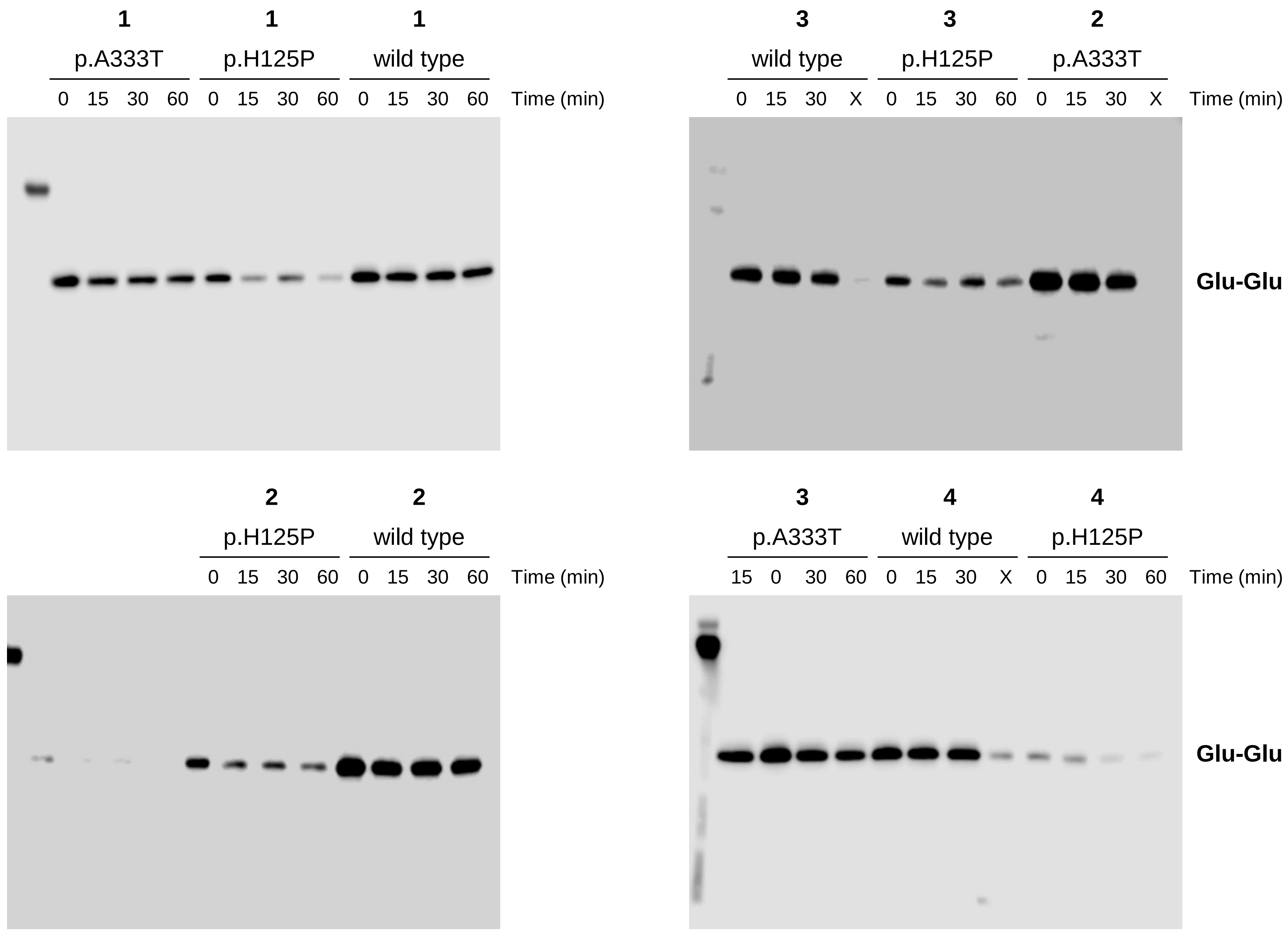

ER stress markers after transient transfection of SCD1 missense mutants in HEK293T cells I.

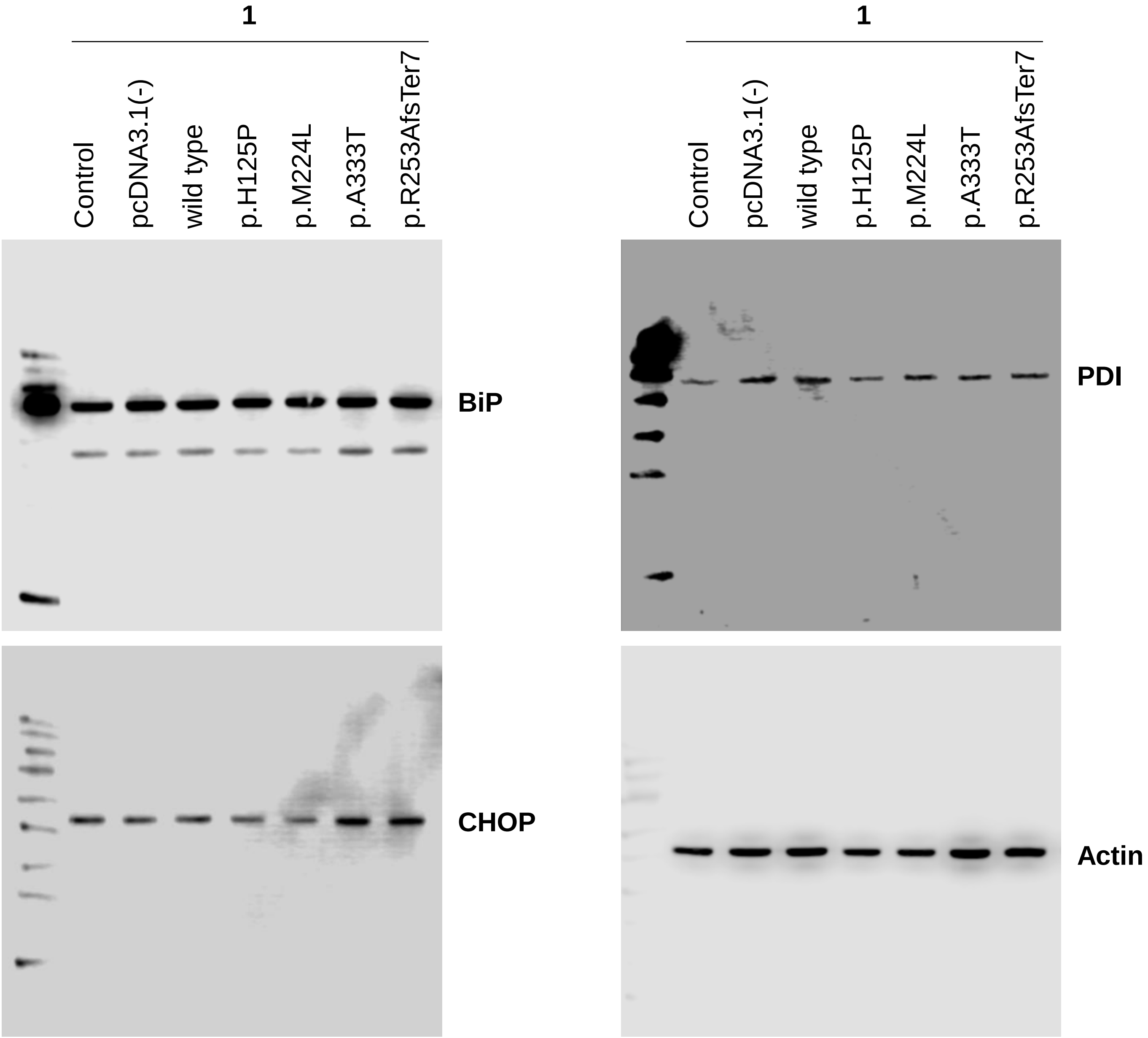

ER stress markers after transient transfection of SCD1 missense mutants in HEK293T cells II.

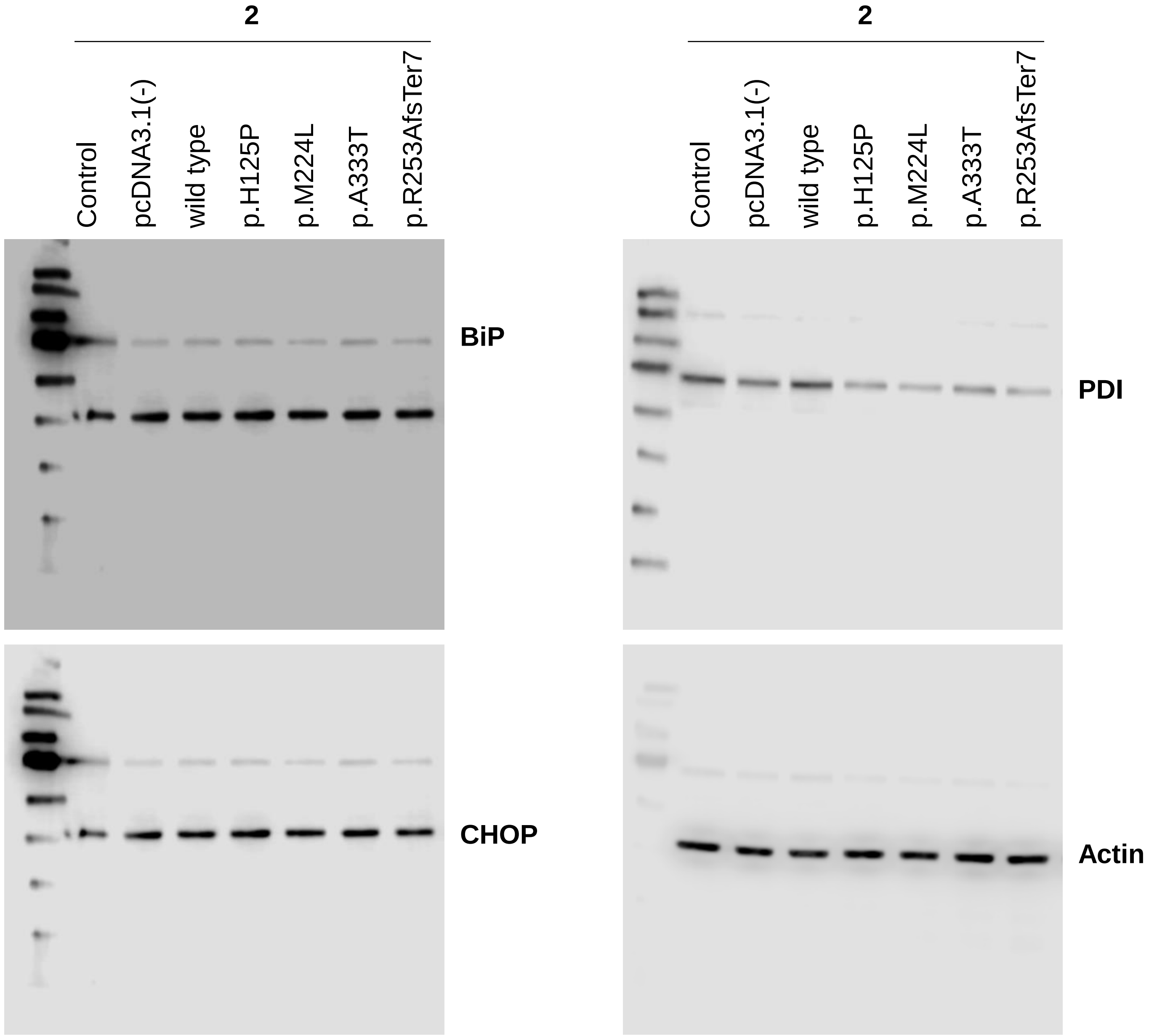

ER stress markers after transient transfection of SCD1 missense mutants in HEK293T cells III.

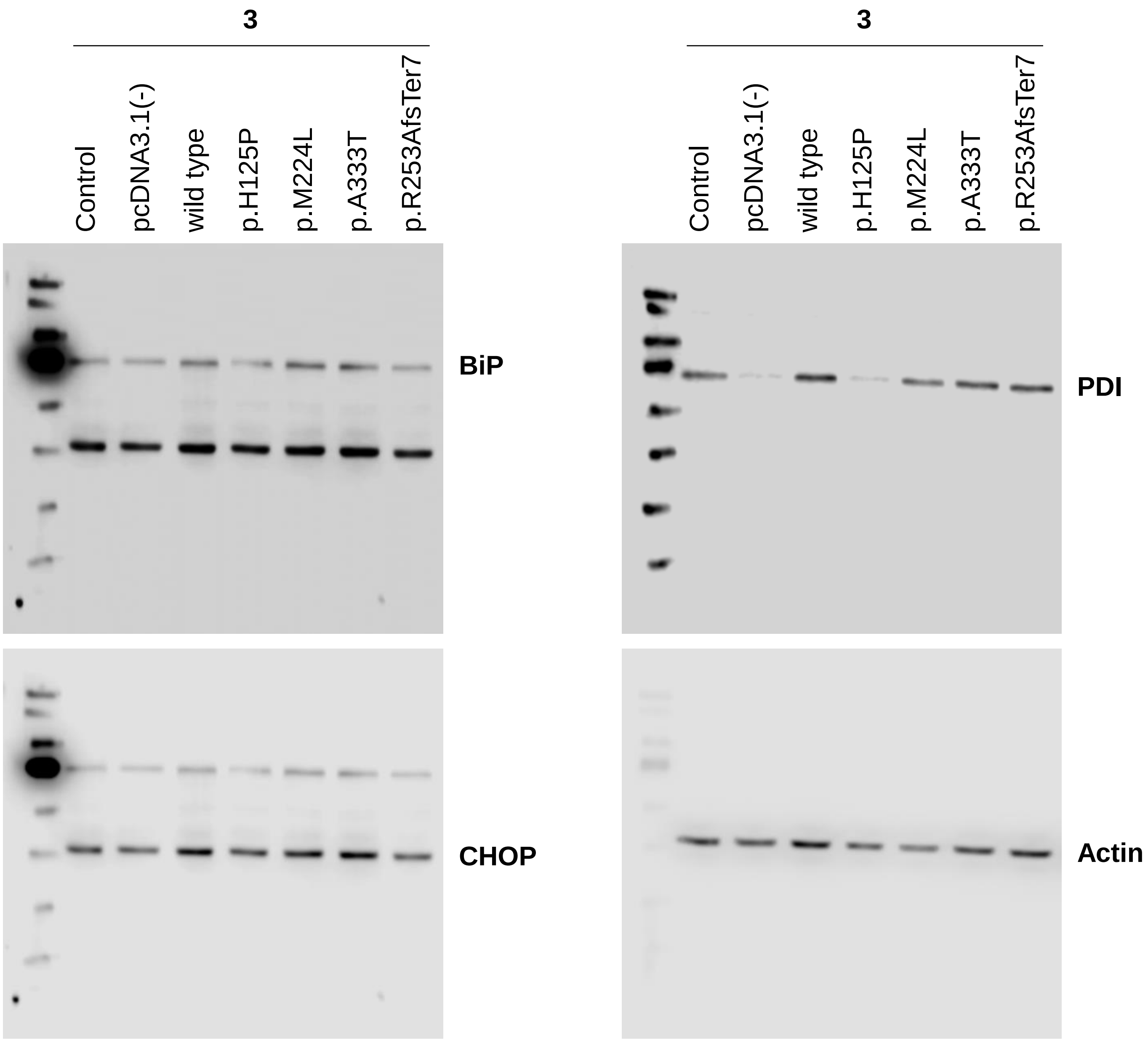

SCD1 protein content of pellet and supernatant from transiently transfected HEK293T cell lysates

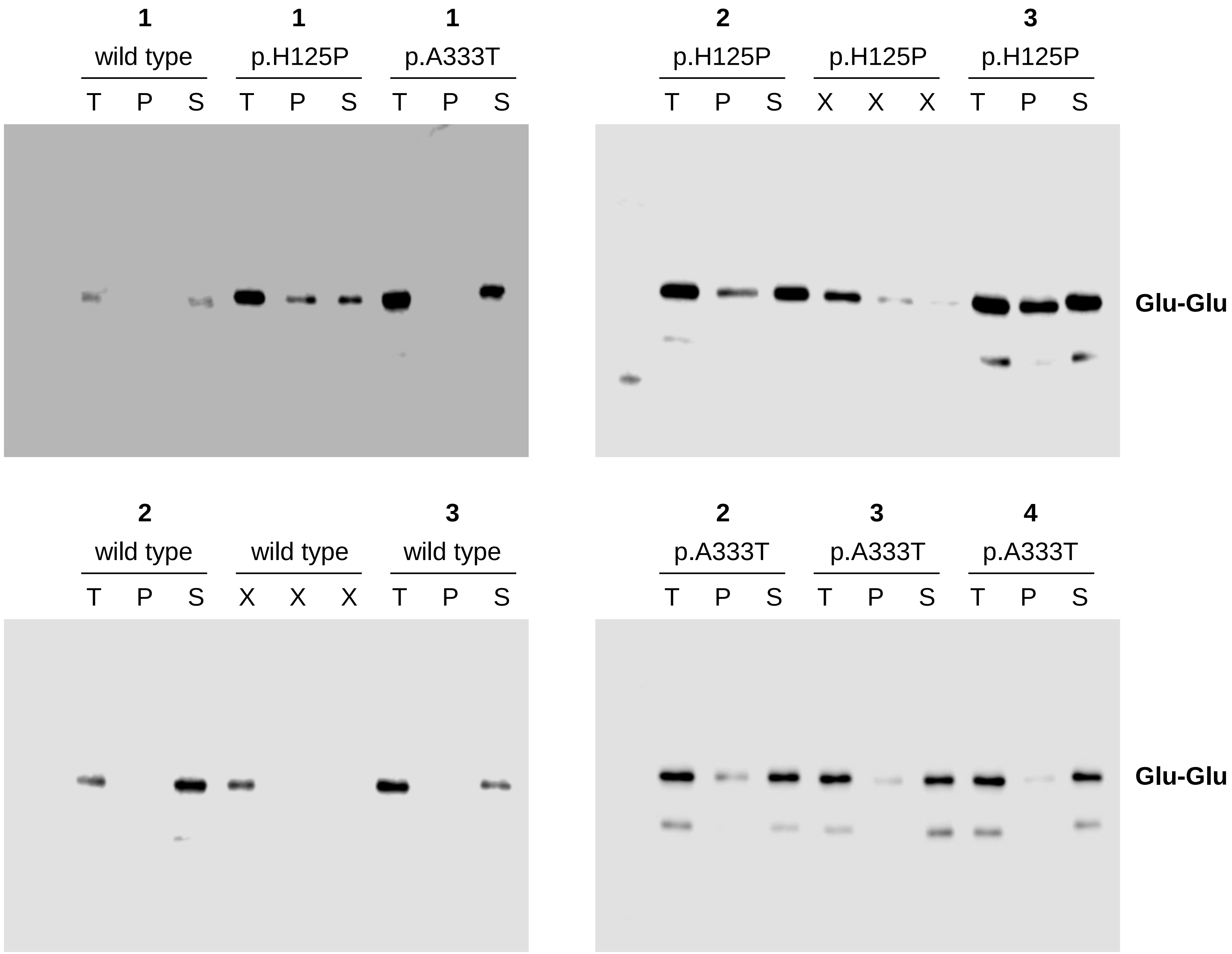

Supplement: Supplementary file 1 [file nutrients-16-03259-s001.zip › nutrients-3199599-supplementary.pdf]
